# Agro-physiological and transcriptome profiling reveal key genes associated with potato tuberization under different nitrogen regimes in aeroponics

**DOI:** 10.1371/journal.pone.0320313

**Published:** 2025-03-28

**Authors:** Rasna Zinta, Jagesh Kumar Tiwari, Tanuja Buckseth, Umesh Goutam, Rajesh Kumar Singh, Ajay Kumar Thakur, Vinod Kumar, Shwetank Singh, Manoj Kumar

**Affiliations:** 1 Indian Council of Agricultural Research-Central Potato Research Institute, Shimla, Himachal Pradesh, India; 2 School of Bioengineering and Biosciences, Lovely Professional University, Phagwara, Punjab, India; 3 Indian Council of Agricultural Research-Indian Institute of Vegetable Research, Varanasi, Uttar Pradesh, India; Jeju National University, KOREA, REPUBLIC OF

## Abstract

Nitrogen (N) is a crucial nutrient for the growth and development of potatoes. However, excessive use of nitrogen fertilizers can have detrimental effects on human health, aquatic ecosystems, and the environment. Therefore, understanding the genes involved in nitrogen metabolism is essential for developing future strategies to improve nitrogen use efficiency (NUE) in plants. This study aimed to identify genes associated with high tuber yield in two contrasting potato varieties Kufri Jyoti (N inefficient) and Kufri Pukhraj (N efficient) grown under low and high nitrogen regimes using an aeroponics system. Both varieties were grown in aeroponics with two nitrogen doses (low N: 0.5 mM N; high N: 5 mM N) using a completely randomized design (CRD) with three replications over two years. The phenotypic results confirmed that Kufri Pukhraj was more nitrogen use efficient compared to Kufri Jyoti, particularly under low nitrogen conditions. Additionally, transcriptome analysis produced high-quality data ( ≥ Q20), ranging from 4.35 to 5.46 Gb per sample. Statistically significant genes (*p* ≤  0.05) were identified based on the reference potato genome. Differentially expressed genes (DEGs) were categorized as either up-regulated or down-regulated in leaf and tuber tissues. Transcriptome profiling of both tuber and leaf tissues revealed genes associated with traits contributing to high tuber yield under both high and low nitrogen conditions. The DEGs were further characterized through gene ontology (GO) annotation and KEGG pathway analysis. Selected genes were validated through real-time quantitative polymerase chain reaction (RT-qPCR) analysis. In summary, several genes were identified as being involved in high tuber yield component traits in potatoes under different nitrogen conditions. These included glutaredoxin, transcription factors (BTB/POZ, AP2/ERF, and MYB), nitrate transporter, aquaporin TIP1;3, glutamine synthetase, aminotransferase, GDSL esterase/lipase, sucrose synthase, UDP-glycosyltransferases, osmotin, xyloglucan endotransglucosylase/hydrolase, and laccases. Additionally, we identified overexpressed genes including cysteine protease inhibitor 1, miraculin, sterol desaturase, and pectinesterase in Kufri Pukhraj under low N stress. Our study highlights these genes’ roles in enhancing tuber yield in potatoes cultivated under both high and low nitrogen in aeroponics.

## Introduction

The potato (*Solanum tuberosum* L.) is the third most important staple food worldwide, following rice and wheat, and it ranks as the most significant non-grain food crop. Potato tubers are a rich source of carbohydrates, vitamins, minerals, and proteins. They also yield high dry matter and calories per unit area and time, making them an excellent source of energy and essential nutrients [1]. As of 2022, global consumption of chemical fertilizers had risen to 187.92 million tons, with nitrogen (N) fertilizers accounting for approximately 58 percent of the total [2]. Nitrogen is a critical nutrient for the growth, yield, and quality of potato tubers. Since potatoes are particularly nitrogen-intensive crops, they require more nitrogen fertilizer than other types of fertilizers [3]. In northern India, for instance, farmers apply high rates of nitrogen fertilizers, ranging from 180 to 280 kg per hectare, to achieve tuber yields of 40 to 50 tonnes per hectare [4]. However, plants only absorb about 40 to 50 percent of the nitrogen applied, meaning that a significant portion is lost to the environment [5]. Excessive use of nitrogen fertilizers can lead to nitrate leaching and greenhouse gas emissions, contributing to soil degradation, human health issues, and air and water pollution. Therefore, it is essential to reduce nitrogen fertilizer use and improve its efficiency to protect the environment.

Improving nitrogen use efficiency (NUE) in plants is a sustainable approach to maintaining production while protecting the environment. NUE is typically measured by a plant’s ability to uptake and utilize nitrogen for tuber production. In potatoes, NUE is estimated as the tuber yield per unit of nitrogen supplied from the soil and fertilizers [6]. Although soil management and agronomic practices have been implemented to optimize nitrogen use in potato fields, thereby reducing the need for nitrogen fertilizers while maintaining yield, progress in breeding more efficient potato varieties has been limited [7–9]. Genotypes with high NUE can produce yields comparable to high-yielding cultivars under nitrogen-limiting conditions and are also responsive to nitrogen when it is available [10]. Therefore, understanding the genes associated with nitrogen-responsive, high-yielding potato varieties is crucial for improving NUE in this crop [11].

Despite the limited research on the genes associated with nitrogen (N) metabolism in potatoes, it is crucial to gather more information on gene expression profiling in N-responsive genotypes under various conditions, whether in the field or controlled environments [12]. Previous studies have identified several genes involved in the nitrogen metabolism process, including those responsible for uptake, translocation, assimilation/utilization, and remobilization. Key genes in this process include nitrate transporters, ammonium transporters, nitrate reductase, nitrite reductase, glutamine synthetase, and asparagine synthetase [13]. The potato genome was sequenced in 2011 [14], and since then, there has been a significant increase in the availability of transcriptomics data in the literature. A few transcriptomic studies have highlighted the genes associated with N metabolism in potatoes [15–17]. Furthermore, multi-omics analyses of excessive nitrogen fertilizer application have identified similar gene networks in potatoes [18]. Until now, most studies have been conducted in field settings, revealing genes that are likely involved in nitrogen use efficiency (NUE) in potatoes. In this study, we explored the NUE in an aeroponic system—a controlled nutrient application method.

The aeroponic system is a soil-less cultivation technology in which nutrient solutions are delivered to the plant root zone through mist or liquid formulation in dark conditions. This technology is particularly useful for producing high-quality minitubers [19]. Similar methods, such as hydroponics [20] and in vitro culture [21], have been employed for screening potato genotypes. We have also demonstrated the application of aeroponics in various potato varieties [22]. In India, two contrasting potato varieties were developed: Kufri Jyoti in the 1960s, which is nitrogen (N) inefficient, and Kufri Pukhraj in 1998, which is nitrogen efficient [23]. Based on field experiments conducted over the years under northern Indian soil conditions, previous studies have concluded that Kufri Pukhraj is more nitrogen-efficient compared to Kufri Jyoti [4]. However, the genetic information regarding the specific genes involved in nitrogen efficiency in this variety under aeroponic conditions remains unclear.

This study aimed to evaluate contrasting potato varieties—Kufri Pukhraj, which is nitrogen efficient, and Kufri Jyoti, which is nitrogen inefficient—under aeroponic conditions. The objective was to confirm differences in phenotypic traits based on agronomic characteristics and to identify genes associated with high yield and nitrogen responsiveness through RNA sequencing. Additionally, selected candidate genes were validated using real-time quantitative polymerase chain reaction (RT-qPCR) analysis. Our findings will provide several potential candidate genes for future research aimed at genetic manipulation to improve nitrogen use efficiency (NUE) in potatoes.

## Materials and methods

### Plant materials and aeroponics cultivation

Two contrasting potato varieties, Kufri Jyoti (nitrogen inefficient) and Kufri Pukhraj (nitrogen efficient), were grown in an aeroponics environment under controlled conditions at the Indian Council of Agricultural Research (ICAR) - Central Potato Research Institute (CPRI) in Shimla, Himachal Pradesh, India (31.1048° N, 77.1734° E, 2,276 m above sea level). These promising varieties were selected based on results from previous long-term field trials. Virus-free in vitro plants of both varieties were multiplied through tissue culture. A total of 10 in vitro plants for each variety were planted in a completely randomized design (CRD) with three replicates during the winter seasons of 2021-22 and 2022-23. The plants were grown in a controlled environment with 11 hours of light and 13 hours of dark, maintaining a day temperature of 23 ±  2°C. They were supplied with high N (5 mM) and low N (0.5 mM) nutrient solutions, following protocols from our previous work [22]. In this study, the high N (5 mM) treatment consisted of various salts, including NH_4_NO_3_ (0.5 mM), Ca(NO_3_)2.4H_2_O (1 mM), KNO_3_ (2 mM), KH_2_PO_4_ (0.5 mM), MgSO_4_.7H_2_O (1 mM), NaCl (0.125 mM), Fe-EDTA (0.0062 mM), H_3_BO_3_ (0.004 mM), MnSO_4_.H_2_O (0.0016 mM), ZnSO_4_.7H_2_O (0.00008 mM), CuSO_4_.5H_2_O (0.00004 mM), Na_2_MoO_4_.2H_2_O (0.00004 mM), CoCl_2_.6H_2_O (0.00004 mM). In comparison, the low nitrogen treatment (0.5 mM) included NH_4_NO_3_ (0.25 mM), KH_2_PO_4_ (0.5 mM), K_2_SO_4_ (1 mM), CaSO_4_.2H_2_O (1 mM), MgSO_4_.7H_2_O (1 mM), NaCl (0.125 mM), Fe-EDTA (0.0062 mM), H_3_BO_3_ (0.004 mM), MnSO_4_.H_2_O (0.0016 mM), ZnSO_4_.7H_2_O (0.00008 mM), CuSO_4_.5H_2_O (0.00004 mM), Na_2_MoO_4_.2H_2_O (0.00004 mM), CoCl_2_.6H_2_O (0.00004 mM). The nutrient solutions were changed every 7 days, and the pH was maintained between 6 and 7 using either H2SO4 or NaOH. The final crop was harvested 110 days after planting. Traits were recorded from at least three plants in each replication. Leaf and tuber samples from both varieties were collected at 60 DAP days after planting. A total of 16 samples (2 varieties ×  2 nitrogen treatments ×  2 plant tissues, i.e., leaf and tuber ×  2 technical replicates for RNA sequencing) were collected in three biological replications. The samples were snap-frozen in liquid nitrogen and stored at -80 °C until further use.

### Agronomic traits evaluation

Plant height (cm) and total leaf area (cm^2^) were measured for each plant at the good vegetative growth stage (60 DAP). The total leaf area per plant was assessed using the LI-3100C Area Meter (LICOR Biosciences, Lincoln, Nebraska, USA). At the harvest stage (110 DAP), the number of tubers, tuber yield, and dry weight of the plant’s shoot, root, and tuber (in grams) were measured. The samples were dried at 70°C for 3 days in an oven (Binder, Tuttlingen, Germany) and then weighed using an electronic balance (Mettler Toledo, Ohio, USA).

### Root morphology

Root morphology was measured on a per-plant basis from at least five plants across three replications in 60 DAP. This was done using the EPSON Expression 12000XL root scanner (Seiko Epson Corporation, Suwa-shi, Nagano-ken, Japan), and the scanned images were analyzed using WinRHIZO Pro 2020a software (Regent Instruments Inc., Quebec, Canada) [24]. The analysis focused on various root traits, including total root length (cm), total root surface area (cm^2^), and root volume (cm^3^), using the software’s default parameters.

### Total chlorophyll content

The total chlorophyll content (in mg per g of fresh weight) was determined in the leaves (specifically, the 4th leaf from the top) of both low-nitrogen (N) and high-nitrogen (N) fed plants at 60 DAP. This was conducted following the procedures outlined by Anderson and Boardman [25]. The absorbance of the chlorophyll was measured at wavelengths of 645 nm and 663 nm, using 80% acetone as a blank, with a UV-1700 Spectrophotometer from Shimadzu Corporation (Kyoto, Japan). The total chlorophyll content was then estimated using the following formulas:


$$TotalChl=\text{20}{\text{.2 (OD}}_{\text{6}4\text{5}})+\text{8}{\text{.02 (OD}}_{\text{663}}\text{) }\times \frac{\text{V}}{\text{1000 }\times W}$$

where, OD_663_ = OD at 663 nm

OD_645_ = OD at 645 nm

V = Total volume of supernatant (ml).

W = Weight of sample (g)

### Total tuber N and NUE parameters.

The total nitrogen (N) content in tubers (measured in grams per plant) was determined on a dry weight basis from tuber tissues at the harvest stage (110 DAP). This was done using the modified Kjeldahl method [26]. At least three plants for each trait per replication were measured. The nitrogen use efficiency (NUE) parameters—namely nitrogen use efficiency, nitrogen uptake efficiency (NUpE), nitrogen utilization efficiency (NUtE), and agronomic NUE (AgNUE)—were estimated based on the total nitrogen content of the plants, as described by Zebarth and colleagues [10].


$$\text{Nitrogen (\%)= }\frac{\text{0}.0014 \times \left(\text{Titre value - Blank value}\right)\text{×100}}{\text{Sample weight }}$$

where, 0.0014 =  factor (i.e., 1 ml of 0.1 N H_2_SO_4_ =  0.0014 g N)


$$\text{Nitrogen use efficiency }\left(\text{NUE}\right)=\frac{\text{Plant dry matter accumulation }}{\text{Crop N supply}}=\text{NUpE }\times \text{NUtE}$$

$$\text{Nitrogen uptake efficiency }\left(\text{NUpE}\right)=\frac{\text{Plant N accumulation }}{\text{Crop N supply}}$$

$$\text{Nitrogen utilization efficiency }\left(\text{NUtE}\right)=\frac{\text{Plant dry matter accumulation }}{\text{Plant N accumulation}}$$

$$\text{Agronomic nitrogen use efficiency }\left(\text{AgNUE}\right)=\frac{\text{Tuber yield }}{\text{Crop N supply}}$$

### Statistical analysis

Data on agronomic and physiological traits, including plant dry weight, tuber yield, total chlorophyll content, root morphology, total tuber nitrogen content, and NUE parameters (NUE, NUpE, NUtE, and AgNUE), were analyzed in three replicates using a two-way ANOVA. This analysis was pooled over the years for two varieties and two nitrogen treatments. An open-source online statistical analysis tool (OPSTAT) (https://opstat.somee.com/) was utilized, and Tukey’s test (*p* ≤ 0.05) was applied to assess homogeneity of variance [27].

### Total RNA isolation and transcriptome analysis

Transcriptome analysis was conducted according to our previous protocols [28]. Total RNA was isolated using a modified CTAB and lithium chloride method [29]. The quality of the isolated RNA was assessed on a 1% denaturing RNA agarose gel and quantified spectrophotometrically using a NanoDrop (ThermoFisher Scientific, Wilmington, Delaware, USA). Paired-end sequencing libraries were prepared using the Illumina TruSeq Stranded mRNA sample prep kit, following the manufacturer’s instructions (Illumina, San Diego, CA, USA). The PCR-enriched libraries were analyzed on the 4200 TapeStation system using high-sensitivity D1000 ScreenTape, as per the manufacturer’s instructions (Agilent Technologies, Santa Clara, CA, USA). The paired-end Illumina libraries were sequenced using the Illumina NextSeq500 platform. The raw data were processed with Trimmomatic v0.38 to obtain high-quality reads (QV >  25). These high-quality reads were then mapped to the reference potato genome [14] using TopHat v2.1.1 software with default parameters [30].

### Differential gene expression analysis

Transcriptome data were assembled using Cufflinks version 2.2.1 software, and differentially expressed genes (DEGs) were analyzed using Cuffdiff version 2.2.1 [30,31]. The analysis focused on four combinations of DEGs: i) Kufri Jyoti (tuber): high N versus low N (control), ii) Kufri Pukhraj (tuber): high N versus low N (control), iii) Kufri Jyoti (leaf): high N versus low N (control), and iv) Kufri Pukhraj (leaf): high N versus low N (control). Log_2_ fold change (FC) values greater than zero were considered up-regulated ( ≥ 2.00), while those less than zero were considered down-regulated ( ≤ -2.00), with a p-value threshold set at 0.05 for statistically significant results. An average linkage hierarchical cluster analysis was conducted on the top 50 DEGs using the Multiple Experiments Viewer (MeV version 4.9.0). Common genes were identified using the Venny 2.1 tool. Scatter plots and volcano plots were created using proprietary R scripts from Eurofins Genomics. Additionally, the combinations of DEGs were analyzed across different varieties for the same tissues, specifically: i) Kufri Pukhraj vs. Kufri Jyoti (low N - tuber), ii) Kufri Pukhraj vs. Kufri Jyoti (high N - tuber), iii) Kufri Pukhraj vs. Kufri Jyoti (low N - leaf), and iv) Kufri Pukhraj vs. Kufri Jyoti (high N - leaf).

### Gene ontology (GO) and KEGG pathways analysis

GO annotations for the DEGs were sourced from the Ensembl Plants database for Solanum tuberosum. The gene counts were categorized into three main Gene Ontology (GO) domains: biological process, cellular component, and molecular function. Bar plots illustrating the distribution of GO terms were created using the WEGO portal (http://wego.genomics.org.cn/cgi-bin/wego/index.pl). Additionally, the functional annotations of the DEGs were performed against the curated KEGG GENES database through the KEGG Automatic Annotation Server (KAAS) (http://www.genome.jp/kegg/ko.html) [32].

### Real time-quantitative polymerase chain reaction (RT-qPCR) analysis

Eight genes, one each from up-regulated and down-regulated groups in leaf and tuber tissues, were analyzed using RT-qPCR. The primers for RT-qPCR were designed with the PrimerQuest Tool from Integrated DNA Technologies (IDT) (see Supplementary Table S6 in S1 File). The cDNA was synthesized using the TaqMan Reverse Transcription Reagent Kit (Applied Biosystems, New Jersey, USA), using the same RNA sample that was employed for RNA sequencing. The RT-qPCR reactions were established with the Power SYBR Green PCR Master Mix on an ABI PRISM HT7900 (Applied Biosystems, Warrington, UK). The temperature and timing profiles for the reaction were as follows: 50 °C for 2 minutes, 95 °C for 10 minutes, followed by 40 cycles of 95 °C for 15 seconds, 60 °C for 1 minute, and 72 °C for 30 seconds. An internal standard, the potato ubiquitin-ribosomal protein gene (ubi3; L22576), was used for normalization, and the data were analyzed in triplicates using the ^∆∆^Ct calculation method [33].

## Results

### Agronomic and physio-biochemical traits

The pooled analysis of variance (ANOVA) for two factors—variety and nitrogen (N)—across the years revealed statistically significant differences (*p* <  0.05) for the variety effect in all traits, except for plant height, tuber number per plant, root length, root volume, and root diameter. Similarly, the nitrogen effect was significant for all traits, except for tuber dry matter, root volume, and root diameter. The interaction between variety and nitrogen was also significant for all traits, including plant height, total leaf area, root dry weight, shoot dry weight, tuber dry matter, tuber number per plant, tuber yield per plant, root length, root surface area, root diameter, tuber nitrogen content, AgNUE, NUE, NUpE, and NUtE, except for total chlorophyll and root volume (Table 1). Therefore, based on the pooled analysis, the average performance of potato varieties under low and high nitrogen treatments in aeroponics is summarized in Table 2.

**Table 1:** Pooled two-way analysis of variance (ANOVA) of two factor Completely Randomized Design (CRD) for years of potato varieties Kufri Jyoti and Kufri Pukhraj grown under high N and low N in aeroponics.

Source of Variation	DF	1. Plant height		2. Total leaf area		3. Root dry weight		4. Shoot dry weight		5. Tuber dry matter		6. Tuber no./ plant	
		SS	MS	SS	MS	SS	MS	SS	MS	SS	MS	SS	MS
Year	1	37.78	37.78	37.05	37.05	0.002	0.002	0.04	0.04	1.16	1.16^**^	8.74	8.74^**^
Variety	1	0.71	0.71	6,255.88	6,255.88^*^	0.32	0.32^**^	10.41	10.41^**^	20.27	20.27^**^	0.61	0.61
Year x Variety	1	60.05	60.05	555.25	555.25	0.01	0.01	1.79	1.79^**^	10.48	10.48^**^	2.11	2.11^*^
Nitrogen	1	3589.04	3589.04^**^	784483	784483.00^**^	7.72	7.72^**^	18.46	18.46^**^	0.01	0.01	199.69	199.69^**^
Year x Nitrogen	1	0.51	0.51	31.14	31.14	0.004	0.004	0.3	0.3	3.01	3.01^**^	1.34	1.34
Variety x Nitrogen	1	335.29	335.29^**^	91786.49	91786.49^**^	0.7	0.70^**^	5.44	5.44^**^	5.56	5.56^**^	50.6	50.60^**^
Year x Variety x Nitrogen	1	44.28	44.28	73.43	73.43	0.01	0.01	1.96	1.96	3.11	3.11^**^	4.29	4.29^**^
Error	12	346.42	28.86	12704.32	1058.69	0.04	0.004	1.2	0.1	0.75	0.06	3.95	0.33
**Source of Variation**	**DF**	**7. Tuber yield/ plant**		**8. Total chlorophyll**		**9. Root length**		**10. Root surface area**		**11. Root volume**		**12. Root diameter**	
		SS	MS	SS	MS	SS	MS	SS	MS	SS	MS	SS	MS
Year	1	32.62	32.62^**^	0.02	0.02	801.83	801.83	18.97	18.97	0.07	0.07	0.001	0.001
Variety	1	19.05	19.05^*^	0.55	0.55^**^	2,660.29	2,660.29	977.75	977.75^*^	0.58	0.58	0.002	0.002
Year x Variety	1	1.62	1.62	0.002	0.002	17,832.44	17,832.44	30.02	30.02	0.01	0.01	0.001	0.001
Nitrogen	1	4903.2	4903.20^**^	2.39	2.39^**^	3664136.9	3664136.90^**^	36390.21	36390.21^**^	4.77	4.77	0.001	0.001
Year x Nitrogen	1	55.07	55.07^**^	0.007	0.007	4,399.33	4,399.33	2.23	2.23	0.15	0.15	0	0
Variety x Nitrogen	1	183.25	183.25^**^	0.007	0.007	7,54,947.04	7,54,947.04^**^	5538.22	5538.22^**^	0.57	0.57	0.006	0.006^*^
Year x Variety x Nitrogen	1	37.11	37.11^**^	0.009	0.009	7,369.66	7,369.66	112.89	112.89	0.003	0.003	0	0
Error	12	27.94	2.32	0.182	0.015	92498.19	7708.18	1774.77	147.89	0.18	0.015	0.01	0.001
**Source of Variation**	**DF**	**13. Tuber N content**		**14. AgNUE**		**15. NUE**		**16. NUpE**		**17. NUtE**			
		SS	MS	SS	MS	SS	MS	SS	MS	SS	MS		
Year	1	0.083	0.083^**^	0.007	0.007^*^	0.375	0.375^**^	0.001	0.001^**^	2.39	2.39		
Variety	1	0.041	0.041^**^	0.586	0.586^**^	0.4	0.4^**^	0	0^**^	3697.43	3697.43^**^		
Year x Variety	1	0.033	0.033^**^	0.085	0.085^**^	0.043	0.043	0	0^**^	12.321	12.321		
Nitrogen	1	0.254	0.254^**^	40.69	40.69^**^	19.548	19.548^**^	0.017	0.017^**^	4607.34	4607.34^**^		
Year x Nitrogen	1	0.095	0.095^**^	0.018	0.018^**^	0.829	0.829^**^	0.001	0.001^**^	100.41	100.41^**^		
Variety x Nitrogen	1	0.172	0.172^**^	0.663	0.663^**^	0.147	0.147^*^	0	0^**^	2849.92	2849.92^**^		
Year x Variety x Nitrogen	1	0.044	0.044^**^	0.1	0.1^**^	0.256	0.256^*^	0	0^**^	120.1	120.10^**^		
Error	12	0.015	0.001	0.018	0.001	0.343	0.029	0	0	74.05	6.17		

Statistical significance:

* *p* <  0.05;

** *p* <  0.01. SS: Sum of Squares; MS: Mean Squares; DF: Degree of Freedom; AgNUE: Agronomic nitrogen use efficiency; NUE: Nitrogen use efficiency; NUpE: Nitrogen uptake efficiency; NUtE: Nitrogen utilization efficiency.

**Table 2:** Mean performance of potato varieties under low and high N treatments in aeroponics based on pooled two-way analysis of variance (ANOVA) of two factor Completely Randomized Design (CRD) for years.

Variety/N		1. Plant height			2. Total leaf area			3. Root dry weight			4. Shoot dry weight			5. Tuber dry matter			6. Tuber no./ plant		
		Yr1	Yr2	Mean	Yr1	Yr2	Mean	Yr1	Yr2	Mean	Yr1	Yr2	Mean	Yr1	Yr2	Mean	Yr1	Yr2	Mean
Kufri Jyoti	High N	100.1	97.44	98.77	1209.45	1215.32	1212.39	1.95	2.06	2.01	4.27	5.7	4.99	18.62	18.28	18.45	18.52	18.76	18.64
	Low N	71.18	62.49	66.84	722.92	731.3	727.11	0.55	0.5	0.53	2.36	2.2	2.28	19.12	15.93	17.53	9.48	10.46	9.97
Kufri Pukhraj	High N	91.83	90.07	90.95	1124.13	1117.85	1120.99	1.48	1.38	1.43	3.12	2.31	2.72	15.21	16.08	15.65	14.5	17.62	16.06
	Low N	72.43	75.5	73.97	892.04	874.13	883.09	0.66	0.62	0.64	1.97	1.86	1.92	16.21	17.1	16.66	12.95	13.44	13.20
CD (5%)	Var.	N/A	N/A		N/A	20.07		0.07	0.06		0.27	0.44		0.22	0.345		N/A	0.734	
	N	7.05	5.19		49.11	20.07		0.07	0.06		0.27	0.44		0.22	0.345		0.58	0.734	
	Var. x N	N/A	7.34		69.45	28.39		0.1	0.09		0.38	0.62		0.31	0.488		0.82	1.039	
CV%	Var.	6.31	4.79		3.73	1.53		4.62	4.37		6.9	11		0.96	1.53		3.14	3.66	
	N	6.31	4.79		3.73	1.53		4.62	4.37		6.9	11		0.96	1.53		3.14	3.66	
	Var. x N	–	–		–	–		–	–		–	–		–	–		–	–	
**Variety/N**		**7. Tuber yield/ plant**			**8. Total Chlorophyll**			**9. Root length**			**10. Root surface area**			**11. Root volume**			**12. Root diameter**		
		Yr1	Yr2	Mean	Yr1	Yr2	Mean	Yr1	Yr2	Mean	Yr1	Yr2	Mean	Yr1	Yr2	Mean	Yr1	Yr2	Mean
Kufri Jyoti	High N	57.56	60.96	59.26	1.96	2.03	2.00	2057.14	2108.09	2082.62	216.3	210.9	213.60	2.14	2.48	2.31	0.2	0.22	0.21
	Low N	23.99	26.3	25.15	1.29	1.37	1.33	928.95	963.91	946.43	103.1	107.58	105.34	1.12	1.1	1.11	0.24	0.27	0.26
Kufri Pukhraj	High N	51.85	59.18	55.52	1.6	1.71	1.66	1770.94	1642.74	1706.84	192.11	199.86	195.99	2.22	2.41	2.32	0.23	0.22	0.23
	Low N	34.31	30.6	32.46	1.07	1.04	1.06	1282.07	1278.11	1280.09	148.34	148.62	148.48	1.77	1.69	1.73	0.2	0.21	0.21
CD (5%)	Var.	2.205	1.154		0.092	0.178		N/A	N/A		N/A	14.62		0.16	0.12		N/A	N/A	
	N	2.205	1.154		0.092	0.178		76.94	120.72		13.39	14.62		0.16	0.12		N/A	N/A	
	Var. x N	3.118	1.632		N/A	N/A		108.81	170.73		18.94	20.68		0.22	0.17		0.047	N/A	
CV%	Var.	3.94	1.95		4.68	8.7		3.82	6.052		6.098	6.588		6.62	4.82		11.52	10.68	
	N	3.94	1.95		4.68	8.7		3.828	6.052		6.098	6.588		6.62	4.82		11.52	10.68	
	Var. x N	–	–		–	–		–	–		–	–		–	–		–	–	
**Variety/N**		**13. Tuber N content**			**14. AgNUE**			**15. NUE**			**16. NUpE**			**17. NUtE**					
		Yr1	Yr2	Mean	Yr1	Yr2	Mean	Yr1	Yr2	Mean	Yr1	Yr2	Mean	Yr1	Yr2	Mean			
Kufri Jyoti	High N	0.44	0.43	0.44	0.31	0.32	0.32	0.1	0.1	0.10	0.002	0.002	0.002	41.69	41.28	41.49			
	Low N	0.52	0.43	0.48	2.5	2.66	2.58	1.91	1.58	1.75	0.052	0.044	0.048	36.16	34.98	35.57			
Kufri Pukhraj	High N	0.17	0.19	0.18	0.28	0.31	0.30	0.08	0.32	0.20	0.001	0.001	0.001	91.35	84.85	88.10			
	Low N	0.76	0.36	0.56	3.4	3.06	3.23	2.62	1.71	2.17	0.077	0.038	0.058	33.28	43.91	38.60			
CD (5%)	Var.	N/A	0.04		0.01	0.06		0.01	N/A		0.005	N/A		2.38	3.27				
	N	0.03	0.04		0.01	0.06		0.01	0.275		0.005	0.004		2.38	3.27				
	Var. x N	0.05	0.06		0.02	0.08		0.02	N/A		0.007	N/A		3.36	4.63				
CV%	Var.	6.19	9.27		0.83	2.83		1.06	22.26		10.77	13.23		3.53	4.8				
	N	6.19	9.27		0.83	2.83		1.06	22.26		10.77	13.23		3.53	4.8				
	Var. x N	–	–		–	–		–	–		–	–		–	–				

Yr1 =  Year 1 (2021-22); Yr2 =  Year 2 (2022-23); CD =  Critical difference; CV =  Coefficient of variation; N =  Nitrogen.

**Fig 1:**
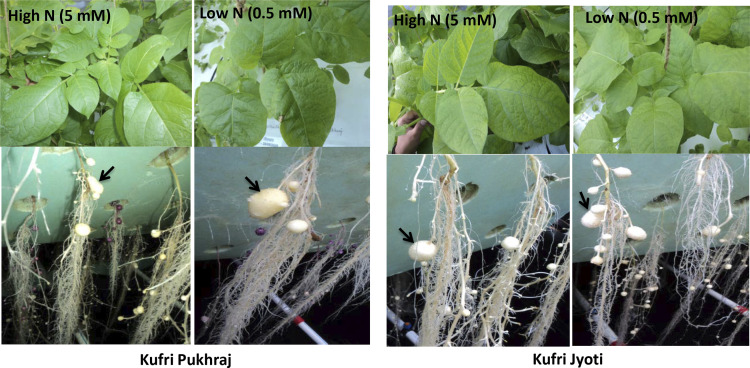
Phenotypic performance of potato varieties Kufri Jyoti and Kufri Pukhraj under high N (5 mM) and low N (0.5 mM) regimes in aeroponics conditions. Arrow indicates tuber formation in these potato varieties.

Statistically significant variations (*p* <  0.05) were observed in two contrasting potato varieties, Kufri Jyoti and Kufri Pukhraj, under high N and low N treatments (Fig 1). Kufri Jyoti exhibited a significantly higher plant height of 98.77 cm compared to Kufri Pukhraj, which measured 90.95 cm under high N conditions. In contrast, under low N conditions, Kufri Jyoti had a plant height of 66.84 cm, while Kufri Pukhraj measured 73.97 cm. Both varieties demonstrated a significantly greater total leaf area under high N compared to low N. In terms of root dry weight, Kufri Jyoti recorded a significantly higher value of 2.01 g than Kufri Pukhraj, which had a root dry weight of 1.43 g under high N. However, under low N, Kufri Pukhraj (0.64 g) showed a significantly higher root dry weight compared to Kufri Jyoti (0.53 g). Shoot dry weight was significantly higher in Kufri Jyoti (4.99 g) than in Kufri Pukhraj (2.72 g) under high N conditions. Tuber dry matter was significantly higher in Kufri Jyoti (18.45%) than Kufri Pukhraj (15.65%) under high N. In contrast, Kufri Pukhraj (16.66%) recorded significantly higher tuber dry matter than in Kufri Jyoti (15.65%) under low N.

The number of tubers per plant was significantly greater in Kufri Jyoti (18.76) under high N, while under low N conditions, Kufri Pukhraj had a significantly higher tuber count of 13.04. Kufri Jyoti also produced a significantly higher tuber yield of 59.26 g/plant compared to Kufri Pukhraj, which yielded 55.52 g/plant under high N. Conversely, under low N, Kufri Pukhraj yielded significantly higher with 32.46 g/plant compared to Kufri Jyoti’s 25.15 g/plant. Overall, the increase in plant biomass—measured in terms of plant height, root and shoot dry weight, total leaf area, tubers per plant, and tuber yield—was greater under high N than under low N for both varieties. However, Kufri Pukhraj produced more tuber yield and a greater number of tubers than Kufri Jyoti under low N supply in aeroponics, indicating that Kufri Pukhraj is an agronomically superior variety under low N conditions. On the other hand, Kufri Jyoti is a higher yielder than Kufri Pukhraj under high N conditions.

Significant variation (*p* <  0.05) was observed in root morphology traits. Under high N conditions, root length was greatest in Kufri Jyoti, measuring 2082.62 cm, compared to Kufri Pukhraj, which measured 1706.84 cm. Conversely, under low N conditions, Kufri Pukhraj recorded a higher root length of 1280.09 cm than Kufri Jyoti, which measured 946.43 cm. Similarly, under high N supply, Kufri Jyoti exhibited a greater root surface area of 213.60 cm^2^ compared to Kufri Pukhraj’s 199.86 cm^2^. However, under low N conditions, Kufri Pukhraj had a higher root surface area of 148.34 cm^2^ than Kufri Jyoti, which had 103.1 cm^2^. In terms of root volume, the values were comparable between Kufri Pukhraj (2.32 cm^3^) and Kufri Jyoti (2.31 cm^3^) under high N. However, under low N, Kufri Pukhraj recorded a higher root volume (1.73 cm^3^) in comparison to Kufri Jyoti (1.11 cm^3^).

NUE is a key parameter for determining nitrogen use efficient varieties. A significant variation (*p* <  0.05) was observed in the total chlorophyll content, tuber nitrogen content, and NUE variables. Total chlorophyll content was significantly higher in high nitrogen conditions compared to low nitrogen in both varieties. Specifically, Kufri Jyoti exhibited significantly higher total chlorophyll levels than Kufri Pukhraj under both high and low nitrogen conditions. Kufri Pukhraj had higher tuber nitrogen content under low nitrogen conditions. AgNUE was significantly higher in Kufri Pukhraj (3.23) than in Kufri Jyoti (2.58) under low nitrogen conditions, whereas it was non-significant in both varieties under high N. Overall, higher NUE was observed in both varieties under low N compared to high N supply. Statistically significant differences showed that higher NUE was recorded in Kufri Pukhraj (2.17) compared to Kufri Jyoti (1.75) under low nitrogen conditions. Importantly, NUtE was significantly higher in Kufri Pukhraj (88.10) than in Kufri Jyoti (41.49) under high N, while non-significant NUtE between Kufri Pukhraj (38.60) and Kufri Jyoti (35.57) under low N. On contrary, NUtE was higher in Kufri Pukhraj than Kufri Jyoti under low N than high N. These results demonstrated that while Kufri Jyoti performed better in terms of tuber yield and its component traits under high nitrogen conditions, whereas Kufri Pukhraj showed better performance under low nitrogen conditions. Thus, Kufri Pukhraj is identified as a nitrogen use efficient variety under low nitrogen supply in aeroponics, based on agronomic, root traits, and NUE parameters. This study confirms that Kufri Pukhraj is a nitrogen use efficient variety suitable for limited nitrogen supply in aeroponics conditions.

### Transcriptome analysis

Tuber and leaf tissues were utilized for RNA sequencing using the Illumina platform, generating high-quality reads ranging from 4.28 to 5.46 Gb per sample. The mapping of these reads to the reference potato genome indicated a similarity of 72.40 to 78.20% (see Supplementary Tables S18 in S1 File). Transcriptome data were assembled using Cufflinks, and DEGs were identified through Cuffdiff software. DEGs were analyzed in the tuber and leaf tissues of the varieties Kufri Jyoti and Kufri Pukhraj under high N conditions compared to low nitrogen (LN) controls (see Supplementary Table S19 in S1 File). DEGs list is given in supplementary files (see Supplementary Excel sheets S1 to S4 in S1 File). Significant DEGs were identified based on the statistical significance (*p* ≤  0.05) and ≥  2 Log_2_ FC) for up-regulated, and ≤  -2 Log_2_ FC values for down-regulated genes.

In the tuber tissues of Kufri Jyoti, when comparing high N conditions to low N (control), a total of 18485 DEGs were identified. Among these, statistically significant (*p* ≤  0.05) 452 genes were up-regulated, while 222 genes were down-regulated. For Kufri Pukhraj, the analysis of tubers under high N versus low N conditions revealed a total of 17344 DEGs, with statistically significant (*p* ≤  0.05) 246 genes up-regulated and 336 down-regulated. The top 20 up-regulated and down-regulated genes in the tuber tissues of both varieties under high N versus low N conditions are presented in Table 3. Similarly, in the leaf tissues of Kufri Jyoti, when comparing high N to low N (control), a total of 17990 DEGs were detected. Out of these, statistically significant (*p* ≤  0.05) 549 genes were up-regulated, while 327 genes were down-regulated. For Kufri Pukhraj’s leaves, a total of 17860 DEGs were identified, with statistically significant (*p* ≤  0.05) 484 genes up-regulated, and 283 genes down-regulated (see Supplementary Table S2 in S1 File). Again, the top 20 up-regulated and down-regulated genes in the leaf tissues of both varieties under high versus low nitrogen conditions are presented in Table 4.

**Table 3:** Selected top differentially expressed genes in tuber tissues of potato varieties under high N versus low N (control) under aeroponics.

Sr. No.	Gene ID	Gene expression (Log_2_ FC)	P value	Gene description
i) Kufri Jyoti (HN vs. LN)				
*Up-regulated*				
1	PGSC0003DMG400028022	5.445	0.002	Sterol desaturase
2	PGSC0003DMG400014459	4.640	0.024	Phosphoethanolamine N-methyltransferase
3	PGSC0003DMG400030212	4.252	0.020	Nitrate reductase
4	PGSC0003DMG400028305	4.210	0.032	Heat shock protein binding protein
5	PGSC0003DMG400013547	4.163	0.000	Sucrose synthase
6	PGSC0003DMG400028396	3.946	0.017	Phosphate transporter PHO1 homolog 10
7	PGSC0003DMG400015229	3.672	0.000	BTB/POZ domain-containing protein
8	PGSC0003DMG402031759	3.638	0.015	Phospholipase A1
9	PGSC0003DMG400004378	3.428	0.046	GDSL esterase/lipase
10	PGSC0003DMG400012479	3.306	0.043	Nitrate transporter
*Down-regulated*				
1	PGSC0003DMG400018505	-3.688	0.038	DAD1
2	PGSC0003DMG400030362	-3.645	0.014	20G-Fe(II) oxidoreductase
3	PGSC0003DMG401026923	-3.369	0.017	1-aminocyclopropane-1-carboxylate oxidase
4	PGSC0003DMG400015129	-3.060	0.001	Defensin protein
5	PGSC0003DMG400021877	-2.860	0.000	Xyloglucan endo-transglycosylase
6	PGSC0003DMG400004109	-2.776	0.000	Xyloglucan endotransglycosylase hydrolase
7	PGSC0003DMG400008000	-2.660	0.001	L-asparaginase
8	PGSC0003DMG400026461	-2.470	0.011	AP2/ERF domain-containing transcription factor
9	PGSC0003DMG400007994	-2.270	0.014	Tuber-specific and sucrose-responsive element binding factor
10	PGSC0003DMG400001418	-2.223	0.003	Transcription factor style2.1
ii) Kufri Pukhraj (HN vs. LN)				
*Up-regulated*				
1	PGSC0003DMG400028182	4.240	0.015	Aquaporin TIP1;3
2	PGSC0003DMG400018286	3.767	0.020	Vetispiradiene synthase
3	PGSC0003DMG400026899	3.591	0.001	Multicystatin
4	PGSC0003DMG400029260	3.008	0.013	Trans-2-enoyl CoA reductase
5	PGSC0003DMG400020388	2.967	0.029	Cationic peroxidase 1
6	PGSC0003DMG400003044	2.702	0.000	Osmotin
7	PGSC0003DMG400013411	2.482	0.000	Chlorophyll a-b binding protein 3C, chloroplastic
8	PGSC0003DMG400023366	2.457	0.000	Quinonprotein alcohol dehydrogenase
9	PGSC0003DMG400003512	2.431	0.034	Laccase
10	PGSC0003DMG400016573	2.343	0.002	Glutaredoxin
*Down-regulated*				
1	PGSC0003DMG400020681	-6.619	0.000	Early nodulin
2	PGSC0003DMG400027976	-5.376	0.003	Hypoxia induced protein conserved region containing protein
3	PGSC0003DMG400027871	-4.769	0.004	RING-H2 finger protein ATL2B
4	PGSC0003DMG400002804	-4.357	0.000	USP
5	PGSC0003DMG400039214	-4.030	0.002	Arachidonic acid-induced DEA1
6	PGSC0003DMG400008000	-4.020	0.017	L-asparaginase
7	PGSC0003DMG400024754	-3.740	0.000	Respiratory burst oxidase homolog protein B
8	PGSC0003DMG400030212	-3.538	0.002	Nitrate reductase
9	PGSC0003DMG400006678	-3.256	0.000	Aspartate aminotransferase
10	PGSC0003DMG400030362	-3.004	0.002	20G-Fe(II) oxidoreductase

DEGs analysis was performed in sample of high N (HN) versus low N (LN, control) of the same variety.

**Table 4:** Selected top differentially expressed genes in leaf tissues of potato varieties under high N versus low N (control) under aeroponics.

Sr. No.	Gene ID	Gene expression (Log_2_ FC)	P value	Gene description
i) Kufri Jyoti (HN vs. LN)				
*Up-regulated*				
1	PGSC0003DMG400005950	7.930	0.000	Multicystatin
2	PGSC0003DMG400018644	6.382	0.020	Protein kinase
3	PGSC0003DMG400026855	6.155	0.009	Endochitinase 4
4	PGSC0003DMG400009513	6.047	0.007	Aspartic protease inhibitor 5
5	PGSC0003DMG400019517	5.510	0.000	Chitin-binding lectin 1
6	PGSC0003DMG400013537	5.451	0.016	Proline-rich protein
7	PGSC0003DMG403020240	4.263	0.006	Glycerophosphodiester phosphodiesterase
8	PGSC0003DMG400004170	4.126	0.007	Asparagine synthetase
9	PGSC0003DMG400018286	3.966	0.003	Vetispiradiene synthase
10	PGSC0003DMG400030784	3.869	0.000	Glutaredoxin family protein
*Down-regulated*				
1	PGSC0003DMG401007615	-5.909	0.000	Sodium/proline symporter
2	PGSC0003DMG400009706	-5.609	0.003	Purine transporter
3	PGSC0003DMG400007683	-4.903	0.006	Sulfate/bicarbonate/oxalate exchanger and transporter sat-1
4	PGSC0003DMG400010050	-4.293	0.000	Proline oxidase/dehydrogenase 1
5	PGSC0003DMG400018129	-3.792	0.031	High-affinity nitrate transport system component
6	PGSC0003DMG400008000	-3.574	0.000	L-asparaginase
7	PGSC0003DMG402010883	-3.556	0.010	MYB transcription factor MYB139
8	PGSC0003DMG400013443	-3.310	0.001	Acyltransferase
9	PGSC0003DMG400009570	-3.284	0.003	MYB transcription factor
10	PGSC0003DMG400009705	-3.002	0.000	Purine transporter
ii) Kufri Pukhraj (HN vs. LN)				
*Up-regulated*				
1	PGSC0003DMG401016475	5.837	0.043	Multicopper oxidase
2	PGSC0003DMG400024755	5.776	0.000	Xyloglucan endotransglucosylase/ hydrolase 1
3	PGSC0003DMG400009514	5.711	0.036	Kunitz-type protease inhibitor
4	PGSC0003DMG400009513	5.642	0.013	Aspartic protease inhibitor 5
5	PGSC0003DMG400026463	5.345	0.000	Aquaporin TIP2;3
6	PGSC0003DMG400003040	4.653	0.002	Osmotin
7	PGSC0003DMG400023620	4.314	0.000	Glutamine synthetase
8	PGSC0003DMG400029201	4.186	0.023	Sesquiterpene synthase 2
9	PGSC0003DMG400010765	4.153	0.023	Glutaredoxin
10	PGSC0003DMG400013815	2.984	0.004	Nitrate transporter
*Down-regulated*				
1	PGSC0003DMG400025967	-5.322	0.001	Pectinesterase
2	PGSC0003DMG400009706	-5.291	0.026	Purine transporter
3	PGSC0003DMG400000110	-5.061	0.008	Wax synthase
4	PGSC0003DMG402010883	-5.022	0.022	MYB transcription factor MYB139
5	PGSC0003DMG400012020	-4.335	0.002	Pectin methlyesterase inhibitor protein 1
6	PGSC0003DMG400000184	-4.151	0.001	Ferric-chelate reductase
7	PGSC0003DMG400019671	-4.134	0.023	Glutaredoxin
8	PGSC0003DMG400031360	-4.083	0.028	UDP-glucoronosyl/UDP-glucosyl transferase family protein
9	PGSC0003DMG400019293	-3.774	0.005	NAC domain-containing protein
10	PGSC0003DMG400026148	-3.728	0.005	USP family protein

DEGs analysis was performed in sample of HN versus low N (LN, control) of the same variety.

**Fig 2:**
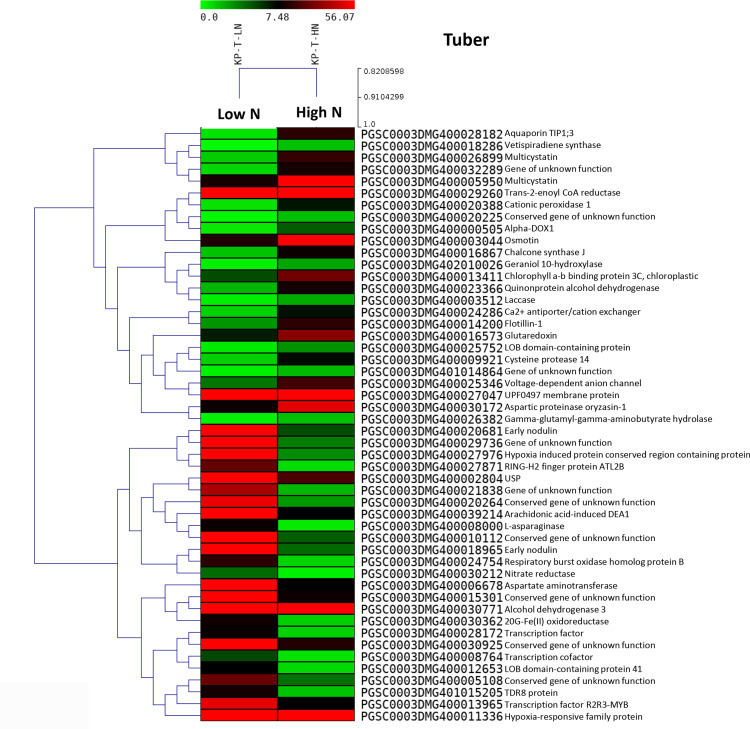
Heat maps of top 50 differentially expressed genes (p < 0.05) in tuber tissues of potato variety Kufri Pukhraj under high N (low N (control)) by RNA-seq. In heat map, each horizontal line refers to a gene. Relatively up-regulated genes are shown in red colour, whereas down-regulated genes are shown in green colour.

**Fig 3:**
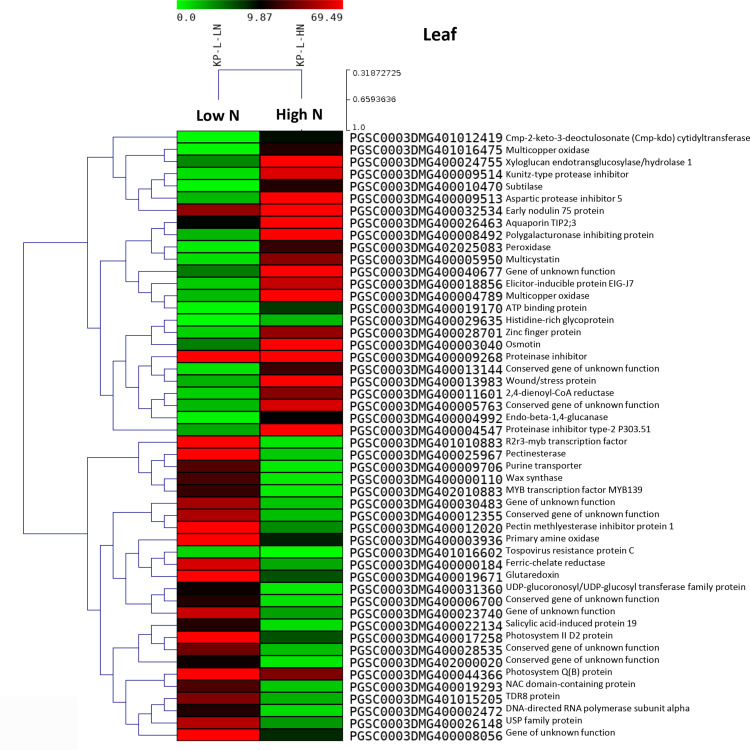
Heat maps of top 50 differentially expressed genes (*p* < 0.05) in leaf tissues of potato variety Kufri Pukhraj under high N (low N (control)) by RNA-seq. In heat map, each horizontal line refers to a gene. Relatively up-regulated genes are shown in red colour, whereas down-regulated genes are shown in green colour.

**Fig 4:**
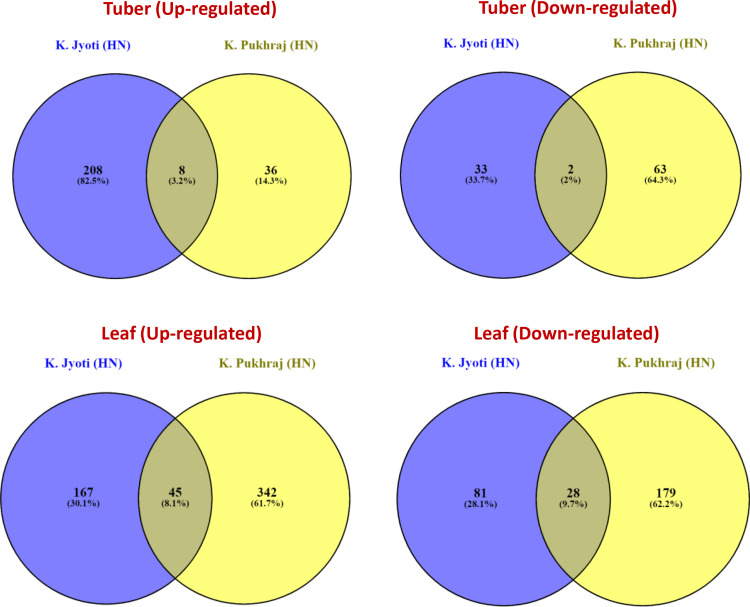
Venn diagrams showing common genes (up-regulated and down-regulated) in potato varieties Kufri Jyoti and Kufri Pukhraj under high N versus (HN) low N (control) (LN) regimes.

Several genes were expressed exclusively in either the control or test samples. A heat map illustrating the top 50 DEGs in the Kufri Pukhraj potato cultivar under high nitrogen (HN) compared to low nitrogen (LN, control) conditions is shown in Fig 2 (tuber) and Fig 3 (leaf). The scatter plot and volcano plot display the significant up-regulated and down-regulated DEGs in both tuber and leaf tissues of Kufri Pukhraj under HN compared to LN (control). A Venn diagram analysis revealed that, in tubers, there were eight up-regulated and two down-regulated genes when comparing Kufri Jyoti (HN) and Kufri Pukhraj (HN) (Fig 4). In contrast, a larger number of common genes were found in leaf tissues, with 45 up-regulated and 28 down-regulated genes identified between Kufri Jyoti (HN) and Kufri Pukhraj (HN) (Fig 4). Another Venn diagram illustrated that in Kufri Jyoti, there were 14 up-regulated and 2 down-regulated genes found in both tuber and leaf tissues. For Kufri Pukhraj, there were 2 up-regulated and 5 down-regulated genes observed between the two tissues (Supplementary Fig S17 in S1 File). Notably, osmotin genes were commonly upregulated in both the tuber and leaf tissues of either Kufri Pukhraj or Kufri Jyoti, indicating their key roles in plant stress responses.

### Identification of potential DEGs

A total of 20 genes that were either up-regulated (Log_2_ FC >  2.0; *p* <  0.05) or down-regulated (Log_2_ FC < -2.0; *p* <  0.05) under high nitrogen conditions (compared to low nitrogen) are summarized in Table 3 (tubers) and Table 4 (leaves). The analysis of DEGs revealed that genes involved in stress response, sugar metabolism, and transcription factors were significantly expressed in both the tuber and leaf tissues of the two potato varieties under high nitrogen compared to the control (low nitrogen). For instance, in the tuber tissues of Kufri Jyoti, several genes were found to be up-regulated, including sterol desaturase (5.44 Log2FC), phosphoethanolamine N-methyltransferase (4.64), and nitrate reductase (4.2). On the other hand, the down-regulated genes in the tuber tissues of Kufri Jyoti included 20G-Fe(II) oxidoreductase (-3.64), 1-aminocyclopropane-1-carboxylate oxidase (-3.36), and xyloglucan endo-transglycosylase (-2.86). Similarly, in the tuber tissues of Kufri Pukhraj, highly up-regulated genes included aquaporin TIP1;3 (4.24), multicystatin (3.59), and trans-2-enoyl CoA reductase (3.0). Conversely, down-regulated genes in the tubers of Kufri Pukhraj included early nodulin (Log2FC -6.61), hypoxia-induced protein (-5.37), and the conserved region containing protein RING-H2 finger protein ATL2B (-4.76).

DEGs were identified in the leaves of both Kufri Jyoti and Kufri Pukhraj. In Kufri Jyoti, several up-regulated genes were found, including multicystatin (7.93), protein kinase (6.38), and endochitinase 4 (6.15). Conversely, the down-regulated genes in the leaf tissues of Kufri Jyoti included a sodium/proline symporter (-5.90), purine transporter (-5.60), and proline oxidase/dehydrogenase 1 (-4.29). In Kufri Pukhraj, up-regulated genes included multicopper oxidase (5.83), xyloglucan endotransglucosylase/hydrolase 1 (5.77), and aquaporin TIP2;3 (5.34). The down-regulated genes in Kufri Pukhraj consisted of pectinesterase (-5.32), purine transporter (-5.29), and MYB transcription factor MYB139 (-5.02). Additional DEGs can be found listed in the supplementary files (see Supplementary Excel sheets S1 to S4 in S1 File).

In this study, we identified nitrogen stress- and nitrogen sufficiency-responsive genes in four combinations of DEGs such as Kufri Pukhraj vs. Kufri Jyoti (control) under low and high nitrogen conditions in tuber and leaf tissues. A complete list of DEGs is available in supplementary files (see Supplementary Excel sheets S13 to S16 in S1 File), and a summary of 10 selected genes for each comparison is presented in Table 5 (for tuber tissues) and Table 6 (for leaf tissues). In the case of tuber tissues under low nitrogen conditions, we observed that some up-regulated genes in Kufri Pukhraj compared to Kufri Jyoti (control) included cysteine protease inhibitor 1, miraculin, and sterol desaturase. Conversely, the down-regulated genes included methylketone synthase Ib and acidic endochitinase, among others (refer to Table 5). Similarly, for leaf tissues under low nitrogen conditions, the up-regulated genes in Kufri Pukhraj compared to Kufri Jyoti (control) included pectinesterase and apyrase 3, while down-regulated genes included zinc finger protein and phenylacetaldehyde synthase (Table 6). A number of genes were also differentially expressed under high nitrogen conditions, as detailed in Tables 5 and 6.

**Table 5:** Selected top differentially expressed genes in tuber tissues of potato varieties Kufri Pukhraj versus Kufri Jyoti (control) under different N regimes in aeroponics.

Sr. No.	Gene ID	Gene expression (Log_2_ FC)	P value	Gene description
i) Low N (Kufri Pukhraj vs. Kufri Jyoti)				
*Up-regulated*				
1.	PGSC0003DMG400010139	12.223	0.006	Cysteine protease inhibitor 1
2.	PGSC0003DMG400010170	10.784	0.008	Miraculin
3.	PGSC0003DMG400028022	6.624	0.006	Sterol desaturase
4.	PGSC0003DMG400010146	6.595	0.000	Kunitz-type tuber invertase inhibitor
5.	PGSC0003DMG400010169	6.303	0.000	Beta-carotene hydroxylase
6.	PGSC0003DMG400010143	6.152	0.000	Cysteine protease inhibitor 1
7.	PGSC0003DMG401021841	5.841	0.038	Replication factor A
8.	PGSC0003DMG401001552	5.463	0.032	3-isopropylmalate dehydratase
9.	PGSC0003DMG400008850	4.839	0.000	Short-chain dehydrogenase
10.	PGSC0003DMG400012032	4.699	0.009	Gamma-gliadin
*Up-regulated*				
1.	PGSC0003DMG400004599	-6.352	0.002	Gene of unknown function
2.	PGSC0003DMG400022355	-5.393	0.034	Gene of unknown function
3.	PGSC0003DMG400025912	-5.169	0.005	Methylketone synthase Ib
4.	PGSC0003DMG400006247	-4.990	0.014	Conserved gene of unknown function
5.	PGSC0003DMG400033882	-4.617	0.009	Acidic endochitinase
6.	PGSC0003DMG400001418	-4.415	0.012	Transcription factor style2.1
7.	PGSC0003DMG401025826	-4.397	0.043	3-hydroxyisobutyryl-CoA hydrolase 1
8.	PGSC0003DMG401029345	-4.208	0.034	Isoform 2 of TMV resistance protein N
9.	PGSC0003DMG400029510	-4.180	0.013	ZFP4 (ZINC FINGER PROTEIN 4)
10.	PGSC0003DMG402005859	-4.084	0.012	Conserved gene of unknown function
ii) High N (Kufri Pukhraj vs. Kufri Jyoti)				
*Up-regulated*				
1.	PGSC0003DMG400010139	8.934	0.000	Cysteine protease inhibitor 1
2.	PGSC0003DMG400010170	7.260	0.000	Miraculin
3.	PGSC0003DMG400010169	6.688	0.001	Beta-carotene hydroxylase
4.	PGSC0003DMG401021841	5.840	0.040	Replication factor A
5.	PGSC0003DMG400005950	5.445	0.000	Multicystatin
6.	PGSC0003DMG400008850	5.249	0.000	Short-chain dehydrogenase
7.	PGSC0003DMG400025168	4.336	0.000	Lipid binding protein
8.	PGSC0003DMG400006800	4.241	0.003	NBS-LRR protein
9.	PGSC0003DMG400016867	4.109	0.016	Chalcone synthase J
10.	PGSC0003DMG400015129	4.043	0.000	Defensin protein
11.	PGSC0003DMG401005482	4.039	0.002	E2F4,5
*Down-regulated*				
1.	PGSC0003DMG400010145	-5.994	0.001	Cysteine protease inhibitor 9
2.	PGSC0003DMG400005683	-5.822	0.019	Gene of unknown function
3.	PGSC0003DMG400026617	-5.386	0.048	Methylketone synthase Ib
4.	PGSC0003DMG402005859	-4.855	0.014	Conserved gene of unknown function
5.	PGSC0003DMG400030212	-4.643	0.008	Nitrate reductase
6.	PGSC0003DMG400004599	-4.320	0.000	Gene of unknown function
7.	PGSC0003DMG400000816	-4.147	0.009	Tospovirus resistance protein A
8.	PGSC0003DMG400017091	-4.055	0.000	Patatin-01
9.	PGSC0003DMG404025785	-4.038	0.037	Dynamin
10.	PGSC0003DMG400003040	-4.025	0.025	Osmotin

DEGs analysis was performed in Kufri Pukhraj versus Kufri Jyoti (control) under low N and high N.

**Table 6:** Selected top differentially expressed genes in leaf tissues of potato varieties Kufri Pukhraj versus Kufri Jyoti (control) under different N regimes in aeroponics.

Sr. No.	Gene ID	Gene expression (Log_2_ FC)	P value	Gene description
i) Low N (Kufri Pukhraj vs. Kufri Jyoti)				
*Up-regulated*				
1.	PGSC0003DMG400002261	5.448	0.005	Conserved gene of unknown function
2.	PGSC0003DMG400025967	5.338	0.000	Pectinesterase
3.	PGSC0003DMG400007335	5.041	0.001	Apyrase 3
4.	PGSC0003DMG401021841	4.781	0.009	Replication factor A
5.	PGSC0003DMG400000292	4.592	0.000	Conserved gene of unknown function
6.	PGSC0003DMG400007385	4.568	0.003	CC-NB-LRR protein
7.	PGSC0003DMG402018893	4.474	0.003	Strictosidine synthase
8.	PGSC0003DMG400009931	4.216	0.036	Zinc-binding family protein
9.	PGSC0003DMG400015225	4.086	0.005	Transposase
10.	PGSC0003DMG401015362	3.926	0.000	ATORC3/ORC3
*Down-regulated*				
1.	PGSC0003DMG400003865	-4.864	0.017	Conserved gene of unknown function
2.	PGSC0003DMG400028701	-4.812	0.001	Zinc finger protein
3.	PGSC0003DMG400024278	-4.411	0.002	Phenylacetaldehyde synthase
4.	PGSC0003DMG400029635	-4.255	0.005	Histidine-rich glycoprotein
5.	PGSC0003DMG400032534	-4.234	0.000	Early nodulin 75 protein
6.	PGSC0003DMG400006247	-4.122	0.000	Conserved gene of unknown function
7.	PGSC0003DMG400002732	-4.106	0.025	VQ motif-containing protein
8.	PGSC0003DMG400004599	-3.971	0.000	Gene of unknown function
9.	PGSC0003DMG400025079	-3.855	0.029	Interferon-induced GTP-binding protein mx
10.	PGSC0003DMG400004259	-3.701	0.029	Thaumatin
ii) High N (Kufri Pukhraj vs. Kufri Jyoti)				
*Up-regulated*				
1.	PGSC0003DMG400020660	5.445	0.047	Protein kinase domain containing protein
2.	PGSC0003DMG400007385	5.080	0.023	CC-NB-LRR protein
3.	PGSC0003DMG400016013	4.840	0.020	Cytochrome P450
4.	PGSC0003DMG402000594	4.707	0.003	Flavonol synthase/flavanone 3-hydroxylase
5.	PGSC0003DMG400029623	4.341	0.015	Salicylic acid/benzoic acid carboxyl methyltransferase
6.	PGSC0003DMG400018924	4.007	0.027	Polyphenol oxidase
7.	PGSC0003DMG400007335	3.961	0.000	Apyrase 3
8.	PGSC0003DMG400007765	3.942	0.027	Sn-1 protein
9.	PGSC0003DMG400011601	3.917	0.005	2,4-dienoyl-CoA reductase
10.	PGSC0003DMG400029503	3.822	0.002	ETAG-A3
*Down-regulated*				
1.	PGSC0003DMG400006247	-5.850	0.002	Conserved gene of unknown function
2.	PGSC0003DMG400030842	-5.484	0.022	PTP-1
3.	PGSC0003DMG400023514	-5.432	0.039	Conserved gene of unknown function
4.	PGSC0003DMG400004599	-4.910	0.001	Gene of unknown function
5.	PGSC0003DMG400019517	-4.671	0.000	Chitin-binding lectin 1
6.	PGSC0003DMG400029085	-4.452	0.000	Mta/sah nucleosidase
7.	PGSC0003DMG400018012	-4.335	0.003	Conserved gene of unknown function
8.	PGSC0003DMG400000110	-4.285	0.016	Wax synthase
9.	PGSC0003DMG400011346	-4.251	0.023	Flowering promoting factor-like 1
10.	PGSC0003DMG400030820	-4.097	0.001	Gene of unknown function

DEGs analysis was performed in Kufri Pukhraj versus Kufri Jyoti (control) under low N and high N.

### GO characterization

**Fig 5:**
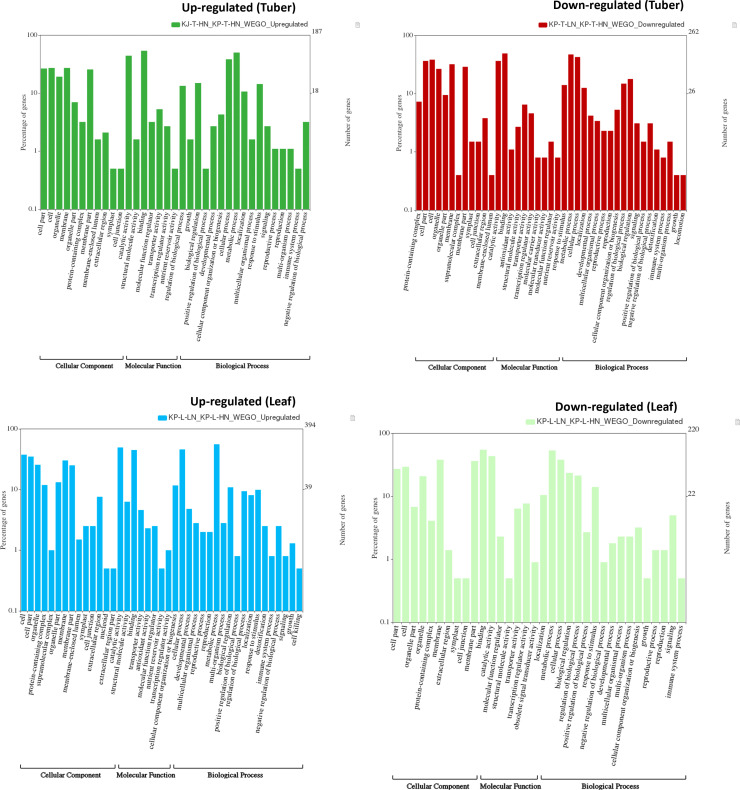
Gene Ontology (GO) characterization for cellular component, molecular function, and biological process of up-regulated and down-regulated DEGs in potato cv. Kufri Pukhraj under high N versus low N (control).

**Fig 6:**
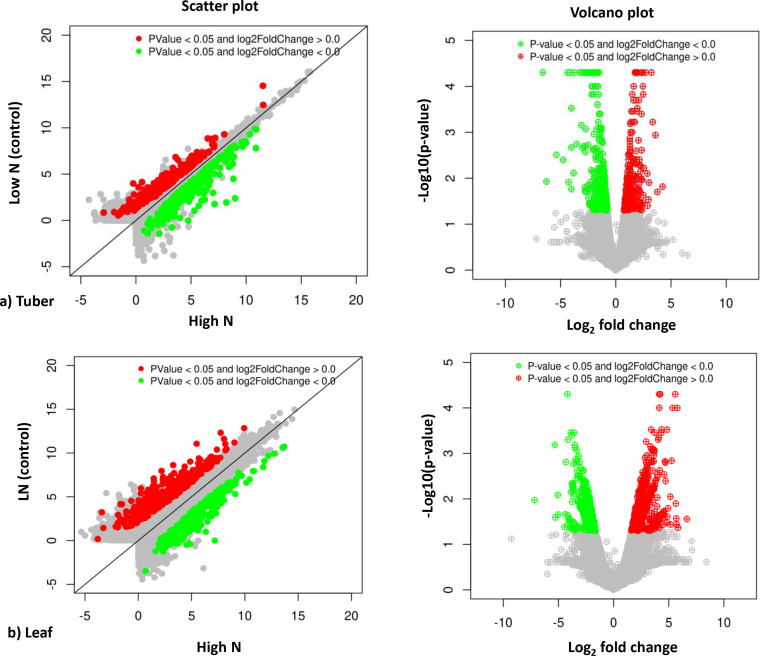
Scatter plot and Volcano plot analysis of up-regulated and down-regulated DEGs in potato cv. Kufri Pukhraj under high N versus low N (control).

DEGs were functionally annotated using Gene Ontology (GO) terms, categorized into three groups: cellular component, molecular function, and biological process (see Supplementary Excel sheets S5 to S8 in S1 File). Among these, the GO term for molecular function had the highest gene count (44096), followed by biological process (36774) and cellular component (32736) in the tuber and leaf tissues of both varieties (see Supplementary Table S20 in S1 File). In the tubers, 21980 molecular function terms were identified, followed by 18141 biological process terms and 16207 cellular component terms. In contrast, in the leaves, 22116 molecular function terms were recorded, followed by 18633 biological process terms and 16529 cellular component terms. Overall, several GO terms, such as cell, cell part, membrane, membrane part, catalytic activity, binding, metabolic process, and cellular process, were found to be highly enriched in both up-regulated and down-regulated DEGs in both tissues (refer to Fig 5). The scatter plot and volcano plot illustrating the genes are shown in Fig 6.

### KEGG pathways analysis

**Fig 7:**
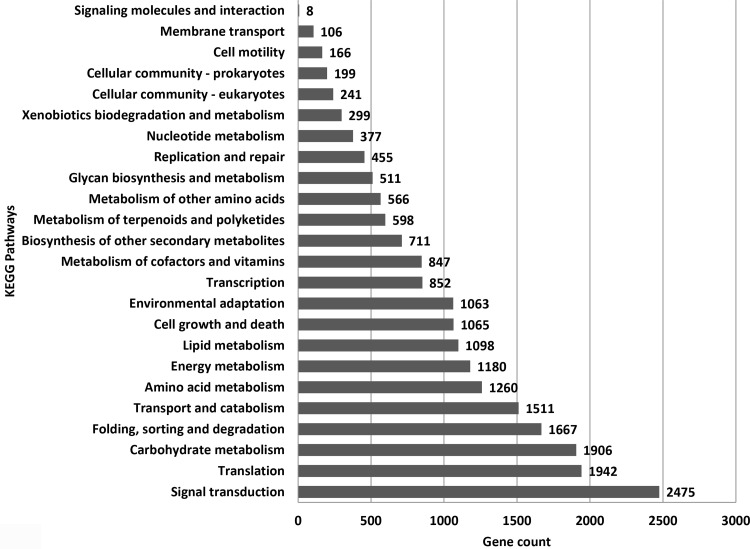
KEGG pathways categorization of up-regulated and down-regulated DEGs in potato cv. Kufri Pukhraj under high N versus low N (control).

DEGs were annotated and categorized into 24 KEGG functional pathways. Out of the total identified 74268 genes, only 21113 genes received KEGG annotations (see Supplementary Excel sheets S9 to S12; Supplementary Table S21 and Table S22 in S1 File). The predominant KEGG pathways represented by these genes in the tissues included signal transduction (2,475 genes), translation (1,942), carbohydrate metabolism (1,906), folding, sorting, and degradation (1,667), transport and catabolism (1,511), amino acid metabolism (1,260), energy metabolism (1,180), lipid metabolism (1,098), and environmental adaptation (1,063) (Fig 7). This highlights the significance of various gene networks involved in nitrogen metabolism, particularly the critical roles of signal transduction and carbohydrate metabolism genes in the growth and development of potato tubers.

### Validation by RT-qPCR analysis

Eight selected genes were validated through RT-qPCR analysis. In tuber tissues, the genes included nitrate reductase (PGSC0003DMG400030212), 20G-Fe(II) oxidoreductase (PGSC0003DMG400030362), aquaporin TIP1;3 (PGSC0003DMG400028182), and the RING-H2 finger protein ATL2B (PGSC0003DMG400027871). In leaf tissues, the genes examined were multicystatin (PGSC0003DMG400005950), sodium/proline symporter (PGSC0003DMG400009706), xyloglucan endotransglucosylase/hydrolase 1 (PGSC0003DMG400024755), and purine transporter (PGSC0003DMG400009706). The gene expression patterns obtained from RT-qPCR aligned with the RNA-seq results; however, slight variations in gene expression were observed (see Supplementary Table S23 in S1 File).

## Discussion

Potato is a crop that requires high nitrogen fertilizer input [4]. Therefore, it is essential to improve NUE at the plant level through the breeding of new cultivars while maintaining yield. This approach will not only save nitrogen, reduce cultivation costs, but also enhance soil and air quality, benefiting the environment and human health [18]. Understanding the genes involved in NUE is crucial for future breeding and biotechnological research. The present study aims to investigate the genes and regulatory elements associated with high tuber yield in potato plants grown under high nitrogen conditions in aeroponics, as well as those with high NUE potential under low nitrogen conditions. Our results identified specific genes in potato leaves and tuber tissues when comparing high nitrogen to low nitrogen environments (control). We observed a significant increase in plant biomass and tuber yield under high nitrogen conditions compared to low nitrogen. Importantly, Kufri Pukhraj exhibited higher NUE than Kufri Jyoti under low nitrogen conditions, confirming previous field-based studies that identified Kufri Pukhraj as a more nitrogen-use-efficient variety than Kufri Jyoti [4]. Our findings align with earlier observations that high nitrogen enhances tuber yield and related traits, while higher NUE is exhibited under low nitrogen conditions [13,17,18,22]. Previous studies have shown that high nitrogen promotes the growth of aboveground plant parts, such as shoots and leaves, while low nitrogen leads to notably smaller potato tubers [48]. Therefore, our study suggests that although high nitrogen increases yield, high NUE is observed under low nitrogen conditions. The NUE-efficient variety, Kufri Pukhraj, has the potential to conserve nitrogen and protect the environment. Additionally, aeroponics technology can be effectively utilized for various studies related to biological development stages, particularly in understanding root biology in the underground parts of potatoes, including roots, stolons, and tubers.

Transcriptome sequencing has become a widely used tool in various crops, including potato, leading to the identification of several regulatory molecules associated with a range of traits. In this study, we identified multiple genes that play crucial roles in plant stress responses and are likely involved in achieving high tuber yields and improving NUE in potatoes grown under an aeroponics system. Notably, the glutaredoxin gene and hormonal signaling molecules were identified as key contributors to high yields and nitrogen metabolism in potatoes. The glutaredoxin family of proteins serves essential functions in plants, including development, defense against stress, redox signaling, hormonal regulation, ion homeostasis, and environmental adaptation [34]. Previous studies have shown that these genes are significant for nitrogen metabolism and tuber development in potatoes [17]. Interestingly, the glutaredoxin gene was upregulated in the leaves and tubers of the Kufri Pukhraj variety, potentially making it more nitrogen-use efficient and resulting in higher yields under nitrogen limitation [17]. Furthermore, it has been demonstrated that overexpression of the CC-type glutaredoxin OsGRX6 impacts hormone signaling and nitrogen status in rice [35]. These findings align with past research that highlights the role of glutaredoxin genes in potato growth and development under varying nitrogen conditions [13]. Additionally, transcription factors (TFs) are important regulatory molecules that play critical roles in plant responses to biotic and abiotic stresses [36]. In this study, transcription factors such as GLK5, MADS64, and bZIP108 were found to be heavily involved in nitrogen metabolism [37]. We observed that a BTB/POZ domain-containing protein TF was upregulated in the tuber tissues of the Kufri Jyoti variety. Similarly, AP2/ERF domain-containing proteins and MYB transcription factors, along with NAC domain-containing TFs, were differentially regulated in our examined varieties. The roles of these genes and transcription factors identified in this study have been supported by previous research indicating their potential in enhancing yield and nitrogen use efficiency in potatoes [37]. Therefore, we suggest that glutaredoxins and transcription factors are likely to play significant roles in achieving high tuber yields and improving NUE in potatoes.

Plant root architecture plays a crucial role in nutrient uptake, particularly through nitrate transporters in potatoes [38,39]. These transporters are essential for regulating nitrogen uptake, root system architecture, protein storage, the source-to-sink relationship, ionic balance, and responses to both biotic and abiotic stresses, as well as maintaining the carbon-nitrogen balance [40]. In this study, we observed that nitrate transporters and nitrate reductase genes were over-expressed in the tubers of Kufri Jyoti and Kufri Pukhraj, highlighting their significant roles in enhancing yield and nitrogen metabolism. Our findings align with previous research that has demonstrated the importance of nitrate transporters in plants [39]. Additionally, ABC transporters were identified in this study, suggesting they may influence the jasmonic acid biosynthesis pathways that regulate potato tuberization [17]. Aquaporins are a diverse family of channel proteins that facilitate the transport of water and small molecules, playing crucial roles in plant development and stress responses [42]. They are essential for the movement of solutes, small molecules, and metal ions in response to both biotic and abiotic stresses in plants. Increasing evidence suggests that aquaporins have important regulatory roles in various processes, including seed germination, tissue expansion, reproductive growth, fruit ripening, water movement, and the maintenance of cellular water homeostasis in plants [41]. In this study, we observed the up-regulation of aquaporin TIP1;3 and aquaporin TIP2;3 genes in tuber and leaf tissues, indicating their role in nitrogen metabolism in potatoes. The involvement of the aquaporin TIP2;1 gene has also been demonstrated in other plant species [43]. Additionally, another crucial category of genes includes glutamine synthetase (GS), which is a key enzyme in the assimilation of inorganic nitrogen into organic forms. In higher plants, there are two forms of GS: cytosolic (GS1) and plastidic (GS2). Among these, GS2 is predominant in most chlorophyll-containing tissues [44]. In the N metabolism group of genes, aminotransferase plays a role in photorespiratory nitrogen assimilation and amino acid biosynthesis. We also found that nitrogen metabolism genes, such as glutamine synthetase (GS), asparagine synthetase, and aspartate aminotransferase, were differentially expressed in our varieties. A previous study showed that the gene encoding alanine-glyoxylate aminotransferase, a peroxisomal enzyme involved in the photorespiratory pathway, increases its expression with nitrogen supplementation. This enzyme catalyzes the conversion of alanine and glyoxylate into glycine and pyruvate [45]. Furthermore, GS is a key component in the nitrogen metabolism biosynthesis pathways associated with potato tuberization [17]. Thus, this study highlights the crucial roles of the nitrate transporter, aquaporins, GS, and L-asparaginase in potato tuber development.

Starch is the primary component of potato tubers. Recently, there has been increased interest in potato starch for both food and non-food applications [46]. In this study, we examined the differential expression of lipid and sugar metabolism-related genes, including sterol desaturase, sucrose synthase, GDSL esterase/lipase genes, and phospholipase, under high nitrogen conditions. Sucrose is the main sugar transported in the phloem tissues of most plants following photosynthesis [17]. Sucrose synthase, a glycosyl transferase enzyme, catalyzes the reversible cleavage of sucrose into fructose and either uridine diphosphate glucose or adenosine diphosphate glucose [48]. The multifunctional role and diversity of the GDSL esterase/lipase gene family have been previously discussed, especially in rice [47]. In our study, we found that sucrose synthase was overexpressed in the tuber tissues, while glycerophosphodiester phosphodiesterase genes were overexpressed in the leaf tissues of the Kufri Jyoti variety. Conversely, the gene for UDP-glucoronyl/UDP-glucosyl transferase family proteins was inhibited in the leaves of the Kufri Pukhraj variety. UDP-glycosyltransferases (UGTs) are crucial enzymes that facilitate the conjugation of sugars with small lipophilic compounds during detoxification and homeostasis processes in plants [49]. These findings align with previous research, which indicated that nitrogen deficiency leads to an increase in sugar content in the stolons, promoting tuberization [17]. Sterols are lipid molecules derived from isoprenoids that play essential roles in eukaryotic cells. Plants produce a complex mixture of sterols that are associated with various functions, including interactions with pathogens and defense responses. In this study, the gene for sterol desaturase was found to be up-regulated in the tuber tissues of the Kufri Jyoti potato variety, suggesting its involvement in tuber growth and development [50]. Additionally, phospholipase enzymes are responsible for catalyzing the hydrolysis of phospholipids and play a vital role in plant metabolism. Among these, phospholipase D is the most significant type in plants, as it is involved in a wide range of functions, including plant growth and development, hormone regulation, and stress responses [51]. Overall, previous studies have highlighted the potential roles of sugar metabolism-related genes in potatoes [49,50]. Therefore, genes related to sugar and lipid metabolism are integral to potato tuber growth and development, influencing yield and nitrogen metabolism.

Stress-responsive genes, heat shock proteins, cell wall proteins, and laccase genes play vital roles in N metabolism under conditions of nitrogen limitation [17]. In this study, the stress-responsive gene osmotin was found to be upregulated in the leaves and tubers of Kufri Pukhraj, while the heat shock protein binding protein and proline-rich protein were upregulated in the tubers and leaves of Kufri Jyoti, respectively. Previous research has also identified similar stress-related genes that promote jasmonic acid biosynthesis in potatoes under nitrogen stress [17]. Additionally, the cell wall is a critical component in plant stress response. The xyloglucan endotransglucosylase/hydrolase multigene family regulates the rebuilding of the cell wall and contributes to stress tolerance in plants [52]. In this study, we observed various cell wall proteins that play diverse roles in potato tuber yield. One notable finding was the upregulation of the xyloglucan endotransglucosylase/hydrolase 1 gene in the leaves of the Kufri Pukhraj variety. Additionally, we identified laccases, which are multi-copper-containing glycoproteins [53]. These enzymes, including nitrite reductase, ascorbate oxidase, and ferroxidase, are involved in catalyzing the oxidation of compounds such as acrylamides and phenols. Our research revealed that laccase and multicopper oxidase genes were significantly induced in both the tubers and leaves of Kufri Pukhraj, indicating their crucial roles in potato tuber development. Previous studies have shown that laccases regulate lignin polymerization and deposition in the plant cell wall in response to environmental stress [54]. In our study of Kufri Pukhraj under low N stress, we identified several overexpressed genes in tuber tissue, including cysteine protease inhibitor 1, miraculin, sterol desaturase, and kunitz-type tuber invertase inhibitor. Additionally, we found that leaf tissues exhibited upregulated genes such as pectinesterase, apyrase 3, strictosidine synthase, zinc-binding family protein, and transposase under low N stress. This suggests that stress response genes are vital in the potato tuberization process and stress adaptation. Furthermore, numerous studies have explored similar gene categories using methods such as genome-wide association studies, metabolomics, and transcriptomics to address drought stress management in rapeseed [55] and meta-QTL analyses for Zn and Fe content analysis in wheat [56].

## Conclusion

**Fig 8:**
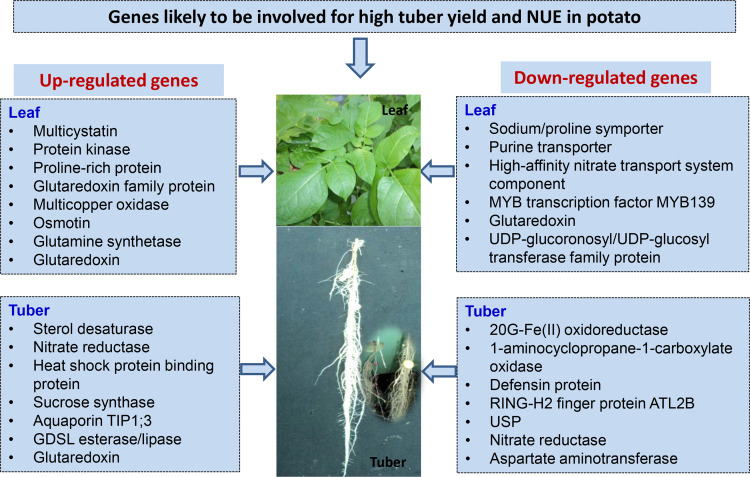
A schematic view of differentially expressed genes likely to be involved in potato above-ground (leaf) and under-ground (tuber) plant parts for up-regulated and down-regulated genes in potato variety Kufri Pukhraj under high N for high tuber yield under aeroponics.

This study reveals that the Kufri Pukhraj variety is nitrogen use efficient under low nitrogen conditions, whereas Kufri Jyoti exhibits high yield under high nitrogen conditions in aeroponics. Transcriptomic profiling has identified several candidate genes that are likely involved in achieving high tuber yields under high nitrogen in aeroponics. These genes include glutaredoxin, various transcription factors (such as BTB/POZ, AP2/ERF, and MYB), a nitrate transporter, aquaporin TIP1;3, glutamine synthetase, aminotransferase, GDSL esterase/lipase, sucrose synthase, UDP-glycosyltransferases, osmotin, xyloglucan endotransglucosylase/hydrolase, and laccases. These genes may be associated with high tuber yield in the potato varieties Kufri Jyoti and Kufri Pukhraj, as well as nitrogen stress management under varying nitrogen conditions in aeroponics. Fig 8 presents a schematic view of genes that are likely involved in the above-ground (leaf) and below-ground (tuber) parts of the potato plant. This includes both up-regulated and down-regulated genes in the Kufri Pukhraj variety under high nitrogen conditions for optimal tuber yield in aeroponics. Further research is necessary to validate these findings with field-level results and to functionally characterize the candidate genes related to high yield and NUE in plants. Overall, this study provides valuable insights into the potential genes involved in tuber yield and NUE in potatoes subjected to high versus low nitrogen conditions in aeroponics.

## Supporting information

S1 File **Suppl. Excel sheet S1.** DEGs in Kufri Jyoti for High N vs Low N (Tuber tissue). **Suppl. Excel sheet S2.** DEGs in Kufri Pukhraj for High N vs Low N (Tuber tissue). **Suppl. Excel sheet S3.** DEGs in Kufri Jyoti for High N vs Low N (Leaf tissue). **Suppl. Excel sheet S4.** DEGs in Kufri Pukhraj for High N vs Low N (Leaf tissue). **Suppl. Excel sheet S5.** Gene Ontology (GO) in Kufri Jyoti for High N vs Low N (Tuber tissue). **Suppl. Excel sheet S6.** Gene Ontology (GO) in Kufri Pukhraj for High N vs Low N (Tuber tissue). **Suppl. Excel sheet S7.** Gene Ontology (GO) in Kufri Jyoti for High N vs Low N (Leaf tissue). **Suppl. Excel sheet S8.** Gene Ontology (GO) in Kufri Pukhraj for High N vs Low N (Leaf tissue). **Suppl. Excel sheet S9.** KEGG pathways in Kufri Jyoti for High N vs Low N (Tuber tissue). **Suppl. Excel sheet S10.** KEGG pathways in Kufri Pukhraj for High N vs Low N (Tuber tissue). **Suppl. Excel sheet S11.** KEGG pathways in Kufri Jyoti for High N vs Low N (Leaf tissue). **Suppl. Excel sheet S12.** KEGG pathways in Kufri Pukhraj for High N vs Low N (Leaf tissue). **Suppl. Excel sheet S13.** DEGs in Kufri Pukhraj vs Kufri Jyoti under Low N (Tuber tissue). **Suppl. Excel sheet S14.** DEGs in Kufri Pukhraj vs Kufri Jyoti under High N (Tuber tissue). **Suppl. Excel sheet S15.** DEGs in Kufri Pukhraj vs Kufri Jyoti under Low N (Leaf tissue). **Suppl. Excel sheet S16.** DEGs in Kufri Pukhraj vs Kufri Jyoti under High N (Leaf tissue). **Suppl. Fig. S17.** Venn diagrams showing common genes (up-regulated and down-regulated) between tuber and leaf tissues of Kufri Jyoti and Kufri Pukhraj. **Suppl. Table S18.** RNA-seq data summary and reference mapping with the Potato genome. **Suppl. Table S19.** DEGs summary in high N vs. low N. **Suppl. Table S20.** GO annotation summary in high N vs. low N. **Suppl. Table S21.** KEGG Annotation Statistics of DEG in high N vs. low N. **Suppl. Table S22.** KEGG Pathway classification of DEG in high N vs. low N. **Suppl. Table S23.** Validation of selected genes through RT-qPCR analysis in high N vs. low N.
(ZIP)
