# Supplementary material for: Agro-physiological and transcriptome profiling reveal key genes associated with potato tuberization under different nitrogen regimes in aeroponics
Source: PLoS One. 2025 Mar 28;20(3):e0320313. doi: 10.1371/journal.pone.0320313 (PMC11952238; doi:10.1371/journal.pone.0320313)
Supplement: S1 File — Suppl. Excel sheet S1. DEGs in Kufri Jyoti for High N vs Low N (Tuber tissue). Suppl. Excel sheet S2. DEGs in Kufri Pukhraj for High N vs Low N (Tuber tissue). Suppl. Excel sheet S3. DEGs in Kufri Jyoti for High N vs Low N (Leaf tissue). Suppl. Excel sheet S4. DEGs in Kufri Pukhraj for High N vs Low N (Leaf tissue). Suppl. Excel sheet S5. Gene Ontology (GO) in Kufri Jyoti for High N vs Low N (Tuber tissue). Suppl. Excel sheet S6. Gene Ontology (GO) in Kufri Pukhraj for High N vs Low N (Tuber tissue). Suppl. Excel sheet S7. Gene Ontology (GO) in Kufri Jyoti for High N vs Low N (Leaf tissue). Suppl. Excel sheet S8. Gene Ontology (GO) in Kufri Pukhraj for High N vs Low N (Leaf tissue). Suppl. Excel sheet S9. KEGG pathways in Kufri Jyoti for High N vs Low N (Tuber tissue). Suppl. Excel sheet S10. KEGG pathways in Kufri Pukhraj for High N vs Low N (Tuber tissue). Suppl. Excel sheet S11. KEGG pathways in Kufri Jyoti for High N vs Low N (Leaf tissue). Suppl. Excel sheet S12. KEGG pathways in Kufri Pukhraj for High N vs Low N (Leaf tissue). Suppl. Excel sheet S13. DEGs in Kufri Pukhraj vs Kufri Jyoti under Low N (Tuber tissue). Suppl. Excel sheet S14. DEGs in Kufri Pukhraj vs Kufri Jyoti under High N (Tuber tissue). Suppl. Excel sheet S15. DEGs in Kufri Pukhraj vs Kufri Jyoti under Low N (Leaf tissue). Suppl. Excel sheet S16. DEGs in Kufri Pukhraj vs Kufri Jyoti under High N (Leaf tissue). Suppl. Fig. S17. Venn diagrams showing common genes (up-regulated and down-regulated) between tuber and leaf tissues of Kufri Jyoti and Kufri Pukhraj. Suppl. Table S18. RNA-seq data summary and reference mapping with the Potato genome. Suppl. Table S19. DEGs summary in high N vs. low N. Suppl. Table S20. GO annotation summary in high N vs. low N. Suppl. Table S21. KEGG Annotation Statistics of DEG in high N vs. low N. Suppl. Table S22. KEGG Pathway classification of DEG in high N vs. low N. Suppl. Table S23. Validation of selected genes through RT-qPCR analysis in high N vs. low N. (ZIP) [file pone.0320313.s001.zip › Suppl. files_updated_20-2-25/Suppl. Figure S17.pptx]

## Slide 1
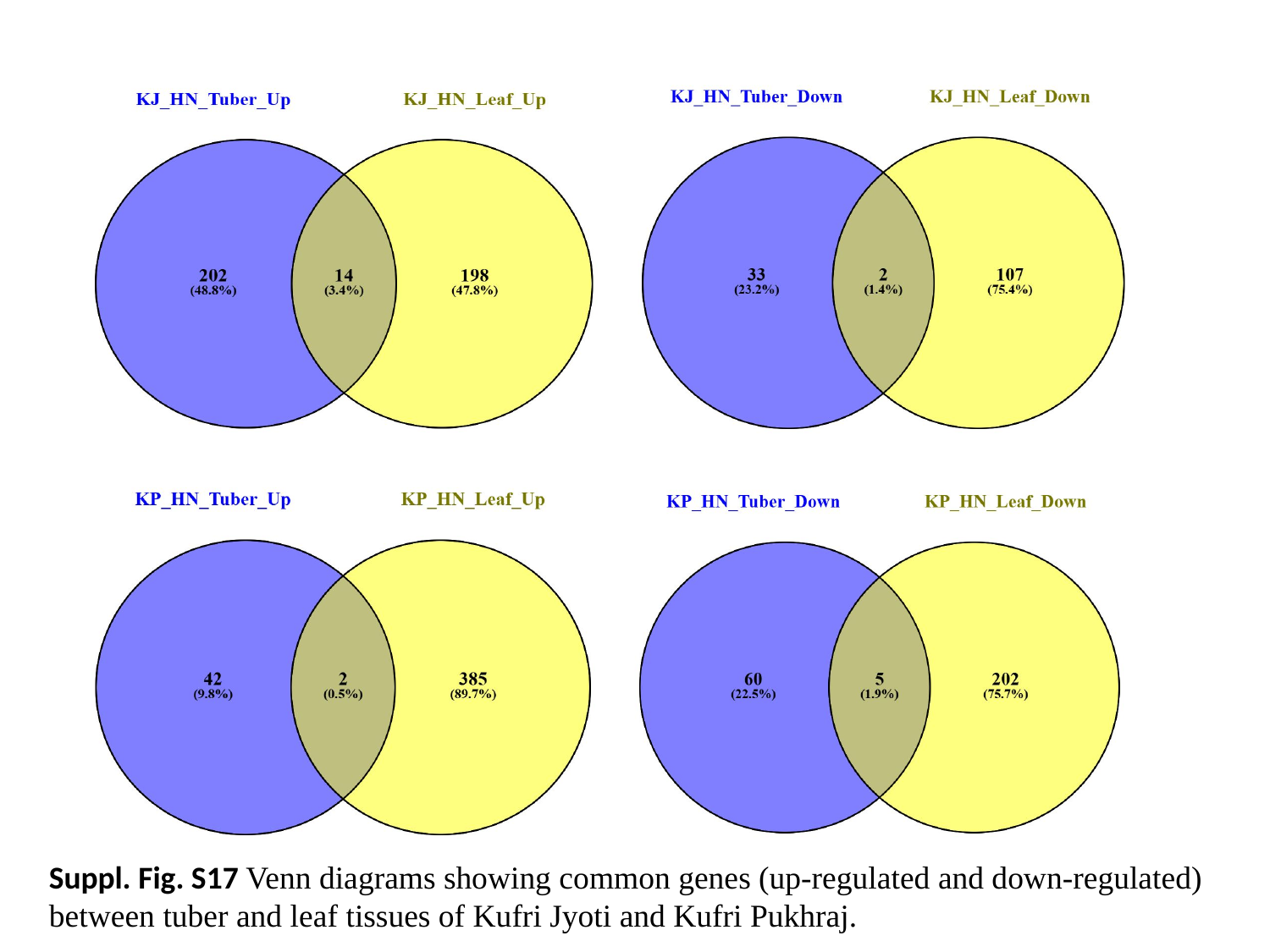

Suppl. Fig. S17 Venn diagrams showing common genes (up-regulated and down-regulated) between tuber and leaf tissues of Kufri Jyoti and Kufri Pukhraj.
