# Supplementary material for: Agro-physiological and transcriptome profiling reveal key genes associated with potato tuberization under different nitrogen regimes in aeroponics
Source: PLoS One. 2025 Mar 28;20(3):e0320313. doi: 10.1371/journal.pone.0320313 (PMC11952238; doi:10.1371/journal.pone.0320313)
Supplement: S1 File — Suppl. Excel sheet S1. DEGs in Kufri Jyoti for High N vs Low N (Tuber tissue). Suppl. Excel sheet S2. DEGs in Kufri Pukhraj for High N vs Low N (Tuber tissue). Suppl. Excel sheet S3. DEGs in Kufri Jyoti for High N vs Low N (Leaf tissue). Suppl. Excel sheet S4. DEGs in Kufri Pukhraj for High N vs Low N (Leaf tissue). Suppl. Excel sheet S5. Gene Ontology (GO) in Kufri Jyoti for High N vs Low N (Tuber tissue). Suppl. Excel sheet S6. Gene Ontology (GO) in Kufri Pukhraj for High N vs Low N (Tuber tissue). Suppl. Excel sheet S7. Gene Ontology (GO) in Kufri Jyoti for High N vs Low N (Leaf tissue). Suppl. Excel sheet S8. Gene Ontology (GO) in Kufri Pukhraj for High N vs Low N (Leaf tissue). Suppl. Excel sheet S9. KEGG pathways in Kufri Jyoti for High N vs Low N (Tuber tissue). Suppl. Excel sheet S10. KEGG pathways in Kufri Pukhraj for High N vs Low N (Tuber tissue). Suppl. Excel sheet S11. KEGG pathways in Kufri Jyoti for High N vs Low N (Leaf tissue). Suppl. Excel sheet S12. KEGG pathways in Kufri Pukhraj for High N vs Low N (Leaf tissue). Suppl. Excel sheet S13. DEGs in Kufri Pukhraj vs Kufri Jyoti under Low N (Tuber tissue). Suppl. Excel sheet S14. DEGs in Kufri Pukhraj vs Kufri Jyoti under High N (Tuber tissue). Suppl. Excel sheet S15. DEGs in Kufri Pukhraj vs Kufri Jyoti under Low N (Leaf tissue). Suppl. Excel sheet S16. DEGs in Kufri Pukhraj vs Kufri Jyoti under High N (Leaf tissue). Suppl. Fig. S17. Venn diagrams showing common genes (up-regulated and down-regulated) between tuber and leaf tissues of Kufri Jyoti and Kufri Pukhraj. Suppl. Table S18. RNA-seq data summary and reference mapping with the Potato genome. Suppl. Table S19. DEGs summary in high N vs. low N. Suppl. Table S20. GO annotation summary in high N vs. low N. Suppl. Table S21. KEGG Annotation Statistics of DEG in high N vs. low N. Suppl. Table S22. KEGG Pathway classification of DEG in high N vs. low N. Suppl. Table S23. Validation of selected genes through RT-qPCR analysis in high N vs. low N. (ZIP) [file pone.0320313.s001.zip › Suppl. files_updated_20-2-25/Suppl. Tables_S18 to S23.docx]

**Suppl.Table S18. RNA-seq data summary and reference mapping with the Potato genome**

| **Sr. No.** | **Sample** | **Raw data** | **Reference mapping** |
| --- | --- | --- | --- |
| **Tuber** | |  |  |
|  | Kufri Jyoti (HN)-R1 | 5.32 Gb | 75.60 % |
|  | Kufri Jyoti (HN)-R2 | 4.28 Gb | 72.40 % |
|  | Kufri Jyoti (LN)-R1 | 5.46 Gb | 72.80 % |
|  | Kufri Jyoti (LN)-R2 | 5.13 Gb | 74.50 % |
|  | Kufri Pukhraj (HN)-R1 | 4.74 Gb | 75.20 % |
|  | Kufri Pukhraj (HN)-R2 | 5.10 Gb | 72.80 % |
|  | Kufri Pukhraj (LN)-R1 | 5.06 Gb | 74.30 % |
|  | Kufri Pukhraj (LN)-R2 | 5.20 Gb | 73.50 % |
|  |  |  |  |
| **Leaf** | |  |  |
|  | Kufri Jyoti (HN)-R1 | 4.35 Gb | 74.60 % |
|  | Kufri Jyoti (HN)-R2 | 4.65 Gb | 78.20 % |
|  | Kufri Jyoti (LN)-R1 | 4.36 Gb | 75.90 % |
|  | Kufri Jyoti (LN)-R2 | 5.10 Gb | 72.60 % |
|  | Kufri Pukhraj (HN)-R1 | 4.66 Gb | 76.80 % |
|  | Kufri Pukhraj (HN)-R2 | 4.80 Gb | 75.10 % |
|  | Kufri Pukhraj (LN)-R1 | 4.50 Gb | 72.80 % |
|  | Kufri Pukhraj (LN)-R2 | 5.20 Gb | 76.50 % |

HN: High N; LN: Low N

**Suppl. Table S19. DEGs summary in high N vs. low N**

| Combination# | Total DEGs | Significant DEGs (*p* < 0.05) | | | |
| --- | --- | --- | --- | --- | --- |
|  |  | Up-regulated | Down-regulated | Exclusive  (Control) | Exclusive  (Treatment) |
| **Tuber** |  |  |  |  |  |
| Kufri Jyoti | 18485 | 452 | 222 | 143 | 497 |
| Kufri Pukhraj | 17344 | 246 | 336 | 173 | 127 |
| **Leaf** |  |  |  |  |  |
| Kufri Jyoti | 17990 | 549 | 327 | 118 | 216 |
| Kufri Pukhraj | 17860 | 484 | 283 | 161 | 280 |

HN: High N, LN: Low N;

DEGs analysis was performed in HN versus LN (control) of the same variety.

**Suppl. Table S20. GO annotation summary in high N vs. low N**

| **Combination#** | **Description** | **Biological Process** | **Cellular Component** | **Molecular Function** |
| --- | --- | --- | --- | --- |
| **Tuber** |  |  |  |  |
| Kufri Jyoti | Down-regulated | 108 | 108 | 135 |
|  | Exclusive control | 170 | 134 | 187 |
|  | Exclusive treated | 31 | 40 | 45 |
|  | Expressed both | 8818 | 7890 | 10759 |
|  | Up-regulated | 281 | 209 | 306 |
| Kufri Pukhraj | Down-regulated | 184 | 156 | 203 |
|  | Exclusive control | 37 | 32 | 36 |
|  | Exclusive treated | 57 | 44 | 55 |
|  | Expressed both | 8320 | 7499 | 10100 |
|  | Up-regulated | 135 | 95 | 154 |
| **Sub-total (Tuber)** | | **18141** | **16207** | **21980** |
| **Leaf** |  |  |  |  |
| Kufri Jyoti | Down-regulated | 189 | 147 | 226 |
|  | Exclusive control | 72 | 62 | 81 |
|  | Exclusive treated | 20 | 30 | 27 |
|  | Expressed both | 8698 | 7760 | 10390 |
|  | Up-regulated | 374 | 292 | 380 |
| Kufri Pukhraj | Down-regulated | 157 | 125 | 187 |
|  | Exclusive control | 115 | 90 | 112 |
|  | Exclusive treated | 46 | 32 | 57 |
|  | Expressed both | 8669 | 7747 | 10334 |
|  | Up-regulated | 293 | 244 | 322 |
| **Sub-total (Leaf)** | | **18633** | **16529** | **22116** |
| **Total (Tuber + Leaf)** | | **36774** | **32736** | **44096** |

HN: High N, LN: Low N;

DEGs analysis was performed in HN versus LN (control) of the same variety.

**Suppl. Table S21. KEGG Annotation Statistics of DEG in high N vs. low N**

| **Sample** | **Identified gene counts** | **KEGG Annotated gene counts** |
| --- | --- | --- |
| **Tuber** |  |  |
| Kufri Jyoti | 19345 | 5397 |
| Kufri Pukhraj | 17863 | 5126 |
| **Leaf** |  |  |
| Kufri Jyoti | 18538 | 5278 |
| Kufri Pukhraj | 18522 | 5312 |
| **Total** | **74268** | **21113** |

HN: High N, LN: Low N;

DEGs analysis was performed in HN versus LN (control) of the same variety.

**Suppl. Table S22. KEGG Pathway classification of DEG in high N vs. low N**

| **KEGG pathways** | **Tuber** | | **Leaf** | |
| --- | --- | --- | --- | --- |
|  | Kufri Jyoti | Kufri Pukhraj | Kufri Jyoti | Kufri Pukhraj |
| **Metabolism** |  |  |  |  |
| Carbohydrate metabolism | 486 | 458 | 481 | 481 |
| Energy metabolism | 291 | 282 | 303 | 304 |
| Lipid metabolism | 282 | 264 | 273 | 279 |
| Nucleotide metabolism | 95 | 95 | 93 | 94 |
| Amino acid metabolism | 321 | 306 | 318 | 315 |
| Metabolism of other amino acids | 150 | 134 | 142 | 140 |
| Glycan biosynthesis and metabolism | 130 | 123 | 130 | 128 |
| Metabolism of cofactors and vitamins | 213 | 201 | 215 | 218 |
| Metabolism of terpenoids and polyketides | 155 | 145 | 146 | 152 |
| Biosynthesis of other secondary metabolites | 204 | 175 | 166 | 166 |
| Xenobiotics biodegradation and metabolism | 84 | 70 | 72 | 73 |
| **Genetic Information Processing** | |  |  |  |
| Transcription | 216 | 214 | 210 | 212 |
| Translation | 494 | 482 | 485 | 481 |
| Folding, sorting and degradation | 426 | 418 | 410 | 413 |
| Replication and repair | 120 | 117 | 105 | 113 |
| **Environmental Information Processing** | |  |  |  |
| Membrane transport | 28 | 27 | 25 | 26 |
| Signal transduction | 631 | 586 | 627 | 631 |
| Signaling molecules and interaction | 2 | 2 | 2 | 2 |
| **Cellular Processes** |  |  |  |  |
| Transport and catabolism | 379 | 370 | 382 | 380 |
| Cell growth and death | 269 | 259 | 266 | 271 |
| Cellular community - eukaryotes | 62 | 60 | 54 | 65 |
| Cellular community - prokaryotes | 50 | 46 | 50 | 53 |
| Cell motility | 41 | 40 | 43 | 42 |
| **Organismal Systems** |  |  |  |  |
| Environmental adaptation | 268 | 252 | 270 | 273 |

HN: High N, LN: Low N;

DEGs analysis was performed in HN versus LN (control) of the same variety.

**Suppl. Table S23. Validation of selected genes through RT-qPCR analysis in high N vs. low N**

| Sr. No | Gene ID | Gene regulation | Gene description | Gene ID | Primer sequence (5’→3’) | Gene expression  (Log_2_ FC) | |
| --- | --- | --- | --- | --- | --- | --- | --- |
|  |  |  |  |  |  | RNA-seq | RT-qPCR |
| **Tuber** | |  |  |  |  |  |  |
| 1. | Kufri Jyoti | Up-regulated | Nitrate reductase | PGSC0003DMG400030212 | F: GTGTAGCTCTCATCCCAAGG  R: TGCCAACAGGTAAGCCTAAG | 4.25 | 3.87 |
| 2. |  | Down-regulated | 20G-Fe(II) oxidoreductase | PGSC0003DMG400030362 | F: CAAAGCACAAAGTACAACCCC  R: AAGACCAGTTTTGAGGCCTAG | -3.64 | -3.08 |
| 3. | Kufri Pukhraj | Up-regulated | Aquaporin TIP1;3 | PGSC0003DMG400028182 | F: GTATTTGCAGGTTCAGGTTCC  R: CCTCCAGAAATGTTAGCCCC | 4.24 | 4.02 |
| 4. |  | Down-regulated | RING-H2 finger protein ATL2B | PGSC0003DMG400027871 | F: CTTTAGGAGGAGCGACAATAGG  R: GGAGTAGCCCTGTTTCTGTTG | -4.76 | -4.30 |
| **Leaf** | |  |  |  |  |  |  |
| 5. | Kufri Jyoti | Up-regulated | Multicystatin | PGSC0003DMG400005950 | F: TTGGGTGAAAGAATGGGAGG  R: AACAGCAAAACGAGCAAGATC | 7.93 | 8.30 |
| 6. |  | Down-regulated | Sodium/proline symporter | PGSC0003DMG400009706 | F: ACTAACCATTCACCAGCCTTC  R: AGAATAAGTTGAGGCAGGAAGG | -5.60 | -4.72 |
| 7. | Kufri Pukhraj | Up-regulated | Xyloglucan endotransglucosylase/ hydrolase 1 | PGSC0003DMG400024755 | F: CACTGCATTTTACCTGTCATCG  R: TCTCTGTTCTCTGTTTCCTTTTCC | 5.77 | 4.39 |
| 8. |  | Down-regulated | Purine transporter | PGSC0003DMG400009706 | F: ACTAACCATTCACCAGCCTTC  R: AGAATAAGTTGAGGCAGGAAGG | -5.29 | -4.21 |

HN: High N, LN: Low N; RT-qPCR analysis was performed in HN versus LN (control) of the same variety.
